# Elucidation of leak-resistance DNA hybridization chain reaction with universality and extensibility

**DOI:** 10.1093/nar/gkaa016

**Published:** 2020-02-05

**Authors:** Shaofei Li, Pan Li, Meihong Ge, Hongzhi Wang, Yizhuang Cheng, Gan Li, Qiang Huang, Huan He, Chentai Cao, Dongyue Lin, Liangbao Yang

**Affiliations:** 1 Center of Medical Physics and Technology, Hefei Institutes of Physical Science, Chinese Academy of Sciences, Hefei, Anhui 230031, China; 2 School of Life Science, Anhui University, Hefei, Anhui 230601, China; 3 Department of Chemistry, University of Science and Technology of China, Hefei, Anhui 230026, China; 4 Cancer Hospital, Chinese Academy of Sciences, Hefei, Anhui 230031, China; 5 State Key Laboratory of Genetic Engineering, School of Life Sciences, Fudan University, Shanghai 200438, China

## Abstract

Hybridization chain reaction (HCR) was a significant discovery for the development of nanoscale materials and devices. One key challenge for HCR is the vulnerability to background leakage in the absence of the initiator. Here, we systematically analyze the sources of leakage and refine leak-resistant rule by using molecular thermodynamics and dynamics, biochemical and biophysical methods. Transient melting of DNA hairpin is revealed to be the underlying cause of leakage and that this can be mitigated through careful consideration of the sequence thermodynamics. The transition threshold of the energy barrier is proposed as a testing benchmark of leak-resistance DNA hairpins. The universal design of DNA hairpins is illustrated by the analysis of hsa-miR-21-5p as biomarker when used in conjunction with surface-enhanced Raman spectroscopy. We further extend the strategy for specific signal amplification of miRNA homologs. Significantly, it possibly provides a practical route to improve the accuracy of DNA self-assembly for signal amplification, and that could facilitate the development of sensors for the sensitive detection of interest molecules in biotechnology and clinical medicine.

## INTRODUCTION

**Figure 1.**
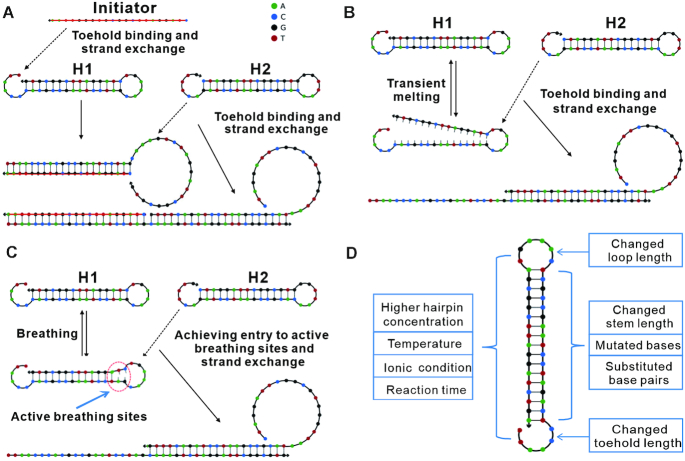
Theoretical schemes for leak-resistant HCR. (**A**) Initiation of the HCR by activation of two hairpins with the initiator strand. Nicked double helix is formed between alternating H1 and H2. (**B** and **C**) Possible pathways for leakage. (B) Transient DNA melting of the stem domain below the melting temperature. The sequestered toehold is completely exposed, and then the reaction is initiated. (C) Achieving entry to active breathing sites next to loop. H2 invades H1 by accessing entry to active breathing sites next to loop. (**D**) Structural framework for the examination of the various factors contributing to leakage. Both hairpin sequences and reaction conditions were programmed from thermodynamic and kinetic perspectives.

By understanding the kinetics and thermodynamics of DNA (1–3), artificially designed molecular systems based on the intrinsic, programmable properties of DNA have become interesting tools in nanotechnology (4). Hybridization chain reaction (HCR) as a DNA self-assembly process was considered to be a significant discovery for the development of functional DNA nanosystems (4,5). Typically, two DNA hairpins (H1 and H2) take part in the HCR (6,7) (Figure 1A). Each one consists of an input domain with an exposed toehold and an output domain with a sequestered toehold. In the presence of the initiator, the hairpins react to form a nicked double helix between alternating H1 and H2 hairpins, amplifying the signal of the initiator (8,9). As a universal post-amplification strategy, HCR can be used for the fabrication of various biosensors (10) and can also be combined with molecular reporters for analytical readouts and bioimaging (6,11–13).

Although it shows substantial promise for biological sensing, the HCR is still plagued by the initiation of the reaction in the absence of the initiator; this is known as leakage (4,14–15). Leakage can critically reduce the sensitivity and scalability because of system background (16). Although a number of methods for reducing leakage have been proposed for DNA strand displacement (4,9,14,16–22), they can be used in only a limited number of contexts and cannot easily guide the rational design of leak-resistant DNA HCR. In a relatively effective approach, a four-point set of guidelines has been established for designing the sequence of hairpin monomers for HCR (15). However, More evidences need to be provided and the explicit guidelines remain lacking (5).

The structural stability and flexibility of DNA molecules are primarily governed by hydrogen bonding (23). Since hydrogen bonds are much weaker than covalent bonds, a double-stranded DNA undergoes breathing fluctuations that spontaneously induce opening and reclosing of the double helix (24), and experiments have shown that DNA breathing is highly localized (25). At higher temperatures, thermal fluctuations can break the base pairing to lead to DNA melting (26). Especially for short DNA duplexes, melting can occur at rather low temperatures to give two separated single strands (23,27). However, besides thermodynamics, the HCR is also a multi-step and multi-state dynamic process of hybridization, fraying, branch migration, etc. Because of the complexity of the problem, there is a lack of sufficient understanding of the nature of leakage in the HCR. Understanding the mechanisms underlying leakage could lead to improved design of HCR systems and enable improved detection schemes for nucleic acid targets.

Surface-enhanced Raman spectroscopy (SERS) has been demonstrated to be an excellent analytical tool owing to its ability to provide rich chemical fingerprint information of analytes and to be conveniently performed under ambient conditions (28,29). In recent years, HCR has been coupled with SERS to provide signal amplification for detectors based on DNA nanotechnology (30–32). Despite these achievements, besides the vulnerability of leakage, current detection schemes are always limited to the specific detection of target homologs, such as miRNA as a new class of biomarkers in cancer diagnosis and therapy (33). The solution of these problems will greatly promote the application of the coupling analytical technique *invivo* or *invitro*.

In this work, we systematically analysed the causes of leakage and demonstrated the leak-resistance mechanism from both thermodynamic and kinetic perspectives. Moreover, we develop a design framework of leak-resistant HCR with universality and extensibility for specific signal amplification of miRNA when used in conjunction with SERS.

## MATERIALS AND METHODS

### Oligonucleotides and RNA of exosomes

DNA and RNA oligonucleotides were synthesized and purified by HPLC (Sangon Biotech Co., China). DNA Hairpins were prepared as monomers at 10 μM in the reaction buffer (20 mM Tris·HCl, 150 mM KCl, 10 mM (NH4)_2_SO_4_, 2.5 mM MgCl_2_, 1% Triton X-100, pH 7.5) using a snap cooling procedure: heating at 95°C for 5 min followed by cooling at 0.5°C min^−1^ to 25°C, and allowing equilibration at room temperature for 30 min before use. The working concentration of DNA was 5 μM, and the initiator was 10 nM in HCR.

Urine samples from bladder cancer patients were obtained from cancer hospital, Chinese academy of sciences, Hefei. Exosomes were isolated with polyethylene glycol (34). The exocrine morphology was characterized by transmission electron microscope (JEM-2100). Size distribution of exosomes was analysised by dynamic light scattering (Zetasizer Nano ZSP). RNA was extracted using a Total Exosome RNA Isolation Kit (Invitrogen). This study was approved by the medical ethics committee of Hefei institute of physical sciences, Chinese academy of sciences and also was performed in accordance with the ethical standards.

### Nanoparticles

Seed gold were synthesized by citrate reduction method. Then, seed solution was mixed with polyvinylpyrrolidone, hydroxylamine hydrochloride and sodium citrate under vigorous stirring. At last, HAuCl_4_ was slowly added, and particles coated with polyvinylpyrrolidone were prepared. The coated gold nanoparticles were characterized by scanning electron microscopy images (SIGMA 500).

### Basic method for hairpin species design

We used hsa-miR-21-5p as a target for illustrative purposes only. The basic framework for two DNA hairpins (HR1 and HR2) was designed as follows (The toehold bases were capitalized, and which in the boxes could be chosen according to the design).

Hsa-miR-21-5p: 5′-uagcuuaucagacugauguuga-3′

DNA sequence corresponding to hsa-miR-21-5p: 5′ - tagcttatcagactgaTGTTGA - 3′

DNA complementary sequence corresponding to hsa-miR-21-5p: 5′ - TCAACAtcagtctgataagcta - 3′

HR1: 5′ - TCAACAtcagtctgataagctaCAAAGTtagcttatcagactga - 3′

HR2: 5′ - tagcttatcagactgaTGTTGAtcagtctgataagctaACTTTG - 3′

### Gel electrophoresis and ethidium bromide staining

The HCR samples were loaded with 10% glycerol onto 12% native polyacrylamide gels prepared with 0.5 × Tris-Borate-EDTA (TBE) buffer. Gels were run at 80 V for 45 min below hybridization temperature. Ethidium bromide was used for gel staining and imaged using a gel imaging system (FluorChem E, ProteinSimple).

### Gel electrophoresis and SERS analysis

Parallel test was used for gel electrophoresis. Nanoparticles were added in the gel loading well 5 min before the electrophoresis finished. After electrophoresis, nanoparticles were closely arranged. Raman spectra were measured on a Lab-RAM HR800 spectrometer with a 633 nm laser. Laser spot was about 0.9 μm in diameter, and the accumulation time was 5 s with a measured power of 0.9 mW. The approach of SERS was illustrated in Supplementary Figure S1.

### NUPACK analysis

DNA sequences were analyzed by NUPACK (http://www.nupack.org/), which was defined in 1 M Na^+^ as a testing benchmark.

### Molecular dynamics simulation

Supplementary Text S1 describes the simulation procedures.

### Theoretical schemes

For two DNA hairpins in HCR, each output domain is sequestered in hairpin. In general terms, we hypothesized that the internal path might be either transient melting of stem domain (Figure 1B) or entry to active breathing sites next to loop (Figure 1C). The former meant that the output domain might be completely exposed and then the strand displacement reaction initiated, whereas the latter meant that the input domain invades the output domain by accessing entry to active breathing sites next to loop in the absence of DNA melting. Based on these, aspects of experiments were programmed for the examination of the factors contributing to leakage (Figure 1D).

## RESULTS

### Effect of stem length on DNA assembly behavior

**Figure 2.**
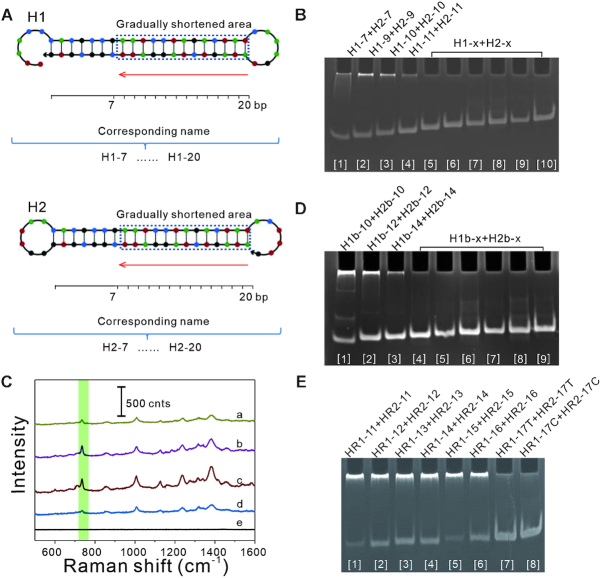
Effect of stem length on DNA assembly behavior. (**A**) Evolution of the hairpin as an example. Stem length was changed in shortened area for assessment leakage. The changed sequence was named depending on stem length. (**B**, **D** and **E**) Native polyacrylamide gel electrophoresis analysis. Leakage was assessed in the reaction buffer for 24 h at 37°C, and leakage products were blocked in the gel loading well. (B) Ten stem lengths based on Dirks and Pierce's design were tested. x represents the values 13, 15 and 17–20. (**C**) SERS analysis for leakage in the gel loading well. The green band indicates the marker of adenine at ∼732 cm^−1^. Spectra a–e correspond to lanes 1–5 in panel B, respectively. (D) Nine reversed stem lengths based on Dirks and Pierce's design were tested. x represents the values 15–20. (E) DNA hairpins designed on the basis of microRNA-21-5p were tested.

We investigated the effect of stem length on DNA assembly behavior. DNA hairpins based on Dirks and Pierce's original design (7) were first evolved by changing stem length (Figure 2A and Supplementary Table S1). Ten stem lengths were separately tested without initiators in the reaction buffer for 24 h at 37°C. Native polyacrylamide gel electrophoresis analysis showed that leakage occurred in the stem lengths of 7–11 bp and leakage products were blocked in the gel loading well (Figure 2B and Supplementary Figure S2A). Stem lengths of 13–20 bp were also tested in response to initiator and showed hybridization products (Supplementary Figure S2B). As a parallel test, SERS was used for readouts (Figure 2C). The marker band of adenine at ∼732 cm^−1^ only appeared in the presence of blocked DNA in the gel loading well, which has been assigned to the ring-breathing mode (35,36).

Considering the different behavior of asymmetric sequences, the stem regions of DNA hairpins based on Dirks and Pierce's original design were reversed, and their length was further changed (Supplementary Figure S3A and Table S2). Nine stem lengths were tested following the same procedure as above. Leakage occurred in the stem lengths of 10–14 bp but not in the longer stem (Figure 2D and Supplementary Figure S3B–D).

The other DNA hairpins based on Dirks and Pierce's original design (7) were also evolved by changing stem length and toehold base composition (Supplementary Table S3). Thirteen pairs of hairpins were tested following the same procedure as above. Native polyacrylamide gel electrophoresis analysis showed that leakage occurred in the stem lengths of 13–14 bp but not in the longer stem (Supplementary Figure S4A).

To further investigate the relationship between stem length and leakage, we are free to select nine miRNAs as initiators, including hsa-let-7a-5p, hsa-miR-18a-5p, hsa-miR-20a-5p, hsa-miR-21-5p, hsa-miR-29a-3p, hsa-miR-30a-5p, hsa-miR-95-3p, hsa-miR-181a-5p and hsa-miR-302a-3p. Twenty-seven pairs of DNA hairpins were designed according to the description in the method (Supplementary Tables S4). In addition, DNA hairpins based on hsa-miR-18a-5p and hsa-miR-181a-5p were also evolved by changing toehold base composition (Supplementary Tables S5). All of these DNA hairpins were estimated following the same procedure as above. For each hairpin species, the results once again showed that the relatively short stems were inclined to cause leakage, whereas the longer stem was the opposite (Supplementary Figure S4B–D). For DNA hairpins designed on the basis of microRNA-21-5p, for instance, leakage occurred in the stem lengths of 11–16 bp but not in the longer (Figures 2E and Supplementary Figure S3D).

From these results, there was a correlation between leakage and length. Owing G-C content and sequence of bases (Supplementary Tables S1–5), the influence of DNA in the same length may be different on leakage, and the transition threshold of length could not be determined for leakage.

### Effect of free energy on DNA assembly behavior

The nearest-neighbor model is one of the extensively used methods to predict the thermostability. NUPACK analysis was carried out for the above hairpin species (Supplementary Tables S1–5). The results suggested that the shorter stem with the higher free energy was inclined to cause leakage whereas the longer stem was the opposite (Supplementary Tables S1–5).

**Figure 3.**
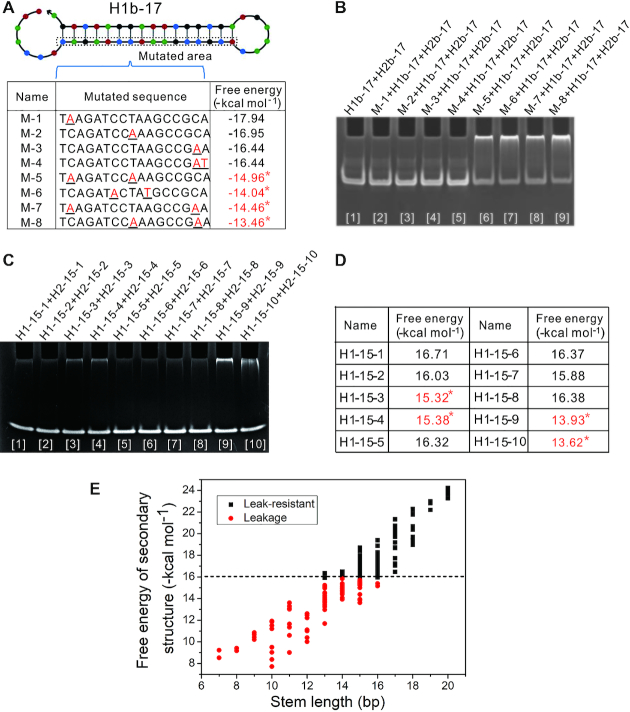
Effect of free energy on DNA assembly behavior. (**A**) Different mutations for H1b-17 under NUPACK analysis. The mutated nucleotides were underlined, and leakage energies were marked with an sterisk. (**B** and **C**) Native polyacrylamide gel electrophoresis assay. Leakage occurred in sequences with higher free energy. Leakage products were blocked in the gel loading well. (B) Leakage for mutated sequences. (C) Leakage for sequences of substituted bases. (**D**) The free energies of substituted sequences. Leakage energies were marked with an steriskin. (**E**) Distribution of free energy for DNA hairpins. The range of the transition threshold maintained between −16.0 and −15.5 kcal mol^−1^.

In order to further verify the effect of free energy on DNA assembly behavior, sequences based on four hairpin species were modified in the stem region by mutation and substitution (Supplementary Table S6) and tested without initiators in the reaction buffer for 24 h at 37°C. Native polyacrylamide gel electrophoresis showed that leakage occurred when the free energy of the secondary structure sequences was increased to a certain extent (Figure 3A–D and Supplementary Figure S5A–D).

In addition, because the free energies are functions of temperature and salt concentration, the three sets of hairpins mentioned in Figure 2 were examined again at 25°C. The results showed that leakage decreased accordingly as the temperature dropped (Supplementary Figure S6A–F). The results indicated that free energy of the secondary structure sequences was critical to determining leakage.

The new G-C pairs contribute −1.5 kcal mol^−1^ of stabilization on average, whereas the A-T pairs add −0.8 kcal mol^−1^ (37). Based on a linear relationship between the free energy of the secondary structure sequence and its length (Supplementary Figure S7A–F), it was possible that there was a transition threshold of free energy against leakage. Owing to the △G^0^ of changing a single nucleotide ranges between 0.06 and 1.90 kcal mol−1 (38), the transition threshold should be in an interval. From DNA hairpins mentioned above, the range of the transition threshold was maintained between −16.0 and −15.5 kcal mol^−1^ (Figure 3E). We proposed that it was possible to resist leakage through a testing benchmark of −16.0 kcal mol^−1^.

To investigate the determinants of leakage, either transient DNA melting or entry to active breathing sites was hypothesized in general terms. Based on the above findings that leakage occurred with the shortening of stem length and the increasing of free energy, it was consistent with the hypothesis of transient DNA melting. On the contrary, the dimers of two DNA hairpin species were not evidently observed (Figure 2B, D and E), which meant that the exposed toehold did not always readily cross with the loop as a sequestered toehold. The remaining possibility was that the exposed toehold could accidentally cross with the loop. In this case, however, the role of each DNA hairpin species in the leakage should be the same with equal opportunity in the absence of active breathing sites, which obviously was in contrast with the results of the experiment (Figure 2B, D and E). In addition, although two base-pairing sites next to the loop for M-4 and H1-18a-01 were exposed by point mutation (Figures 3A and Supplementary Table S6), leakage did not occur (Figure 3B and Supplementary Figure S5A-B). These suggested that DNA strand displacement may not be initiated by entry to active breathing sites. More evidence was provided below.

### Effects of exposed toehold and loop length on DNA assembly behavior

The kinetics of strand displacement has showed rate constant saturation with toehold length for ∼6 nucleotides (nt) (1,39–40). In order to further refute the hypothesis of entry to active breathing next to loop, four hairpin species with 8-nt exposed toehold and 8-nt loop were investigated in the reaction buffer for 24 h at 37°C (Supplementary Table S7), and native polyacrylamide gel electrophoresis analysis showed that no significant leakage occurred (Supplementary Figure S8A). Compared with 6-nt loop, because two additional bases on 8-nt loop were similar to the open ones at the end of stem, the results indirectly suggested that the strand displacement reaction could not occur by entry to active breathing sites.

**Figure 4.**
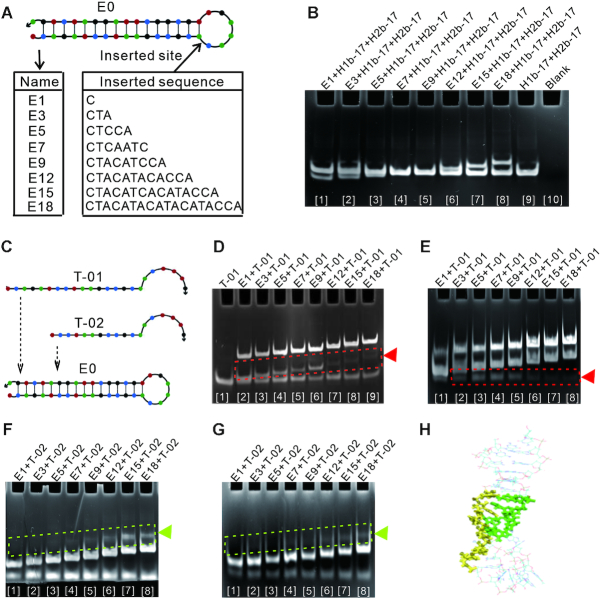
Effect of loop length on DNA assembly behavior. (**A**) The loop of E0 was enlarged by inserting bases. The changed sequence was named depending on the number of bases inserted. (**B**) Native polyacrylamide gel electrophoresis assay for leakage with enlarged hairpin after incubation for 48 h at 37°C. No leakage products were blocked in the gel loading well. (**C**) Two single strands of DNA for assessing the hybridization. (**D**–**G**) Native polyacrylamide gel electrophoresis assay for hybridization. The system was incubated for 48 h at 37°C. T-01 targeted each hairpin species to form dimmers. T-02 only targeted parts of hairpins. (D) T-01 was incubated with each hairpin in the ratio of 3:2. The triangle indicates the consuming hairpins. (E) T-01 was incubated with each hairpin in the ratio of 2:3. The triangle indicates the consuming T-01. (F) T-02 was incubated with each hairpin in the ratio of 3:2. The triangle indicates the formed dimmers. (G) T-02 was incubated with each hairpin in the ratio of 2:3. The triangle indicates the formed dimmers. (**H**) Molecular dynamics simulations for E18. The loop was in stable sequestered state. The molecular conformation of the loop is in bold areas.

Next, loops of three hairpin species based on H1b-17, H1-18a-17b and H1-181a-17b were enlarged by inserting bases. These enlarged loops did not form additional secondary structures (Supplementary S8B and Table S7). Compared with the original sequence, each of the inserted sequences was a lack of the exposed toehold to simplify the study. In the case of E0 based on H1b-17 (Figures 4A), nine loop lengths were incubated separately with H1b-17 and H2b-17 in the reaction buffer at 37°C, and native polyacrylamide gel electrophoresis analysis showed that all sequences maintained good stability against leakage in 24 and 48 h (Figures 4B and Supplementary Figure S9A). For hairpin species based on H1-18a-17b and H1-181a-17b, the same results were obtained (Supplementary Figure S9B). These showed that the strand displacement reaction could not occur by entry to active breathing site, although loops were extended to some extent and the corresponding free energy was increased

In an attempt to explore more visual evidence, two single strands of DNA were used to exclude the possibility of entry to active breathing site (Figure 4C and Supplementary Table S7). T-01 is completely complementary to the loop and stem of E0. Compared with T-01, T-02 is shorter in order to avoid the initial reaction from the naked terminal of hairpin stem. By oligo software version 7.60 and primer 5, both T-01 and T-02 cannot bind to the inserted sequences in E0. T-01 and T-02 incubated separately with the hairpin from E1 to E18 in the ratio of 3:2 and 2:3 at 37°C. The hybridization was analyzed by native polyacrylamide gel electrophoresis at 2, 24 and 48 h (Figure 4D–G and Supplementary Figure S9C–J). In terms of consumption or products, the results showed that both T-01 and T-02 tended to hybridize with hairpins with longer loops in 48 h. Even though T-01 is longer than hairpin stem and the rate constant of hybridization should be dominant (6), the obvious consumption of components occurred in the systems of E12 to E18 (Figure 4D-E and Supplementary Figure S9C–F). Similarly, for T-02, the obvious products were about in the systems of E12 to E18 (Figure 4F-G and Supplementary Figure S9G–J). Furthermore, hairpin species based on H1-18a-17b and H1-181a-17b were also explored as above (Supplementary Table S7), and similar results were showed (Supplementary Figure S10A–F). Combined with the result that hairpins with enlarged loops still had good stability against leakage above, these suggested that even short chains seemed to be not easy to hybridize by entry to active breathing site next to loop in the absence of DNA melting.

We also used molecular dynamics to simulate the states of E18 in a solvent environment. The results showed that the molecular conformation of the loop was stable in the sequestered state, only occasionally exposed and then quickly returned to the buried state (Figure 4H and Supplementary 10G-H). These results may complement the reason why T-01 and T-02 could hybridize with E18.

### Effects of DNA concentration and reaction conditions on DNA assembly behavior

Other factors including DNA concentration, reaction time and ionic liquid dosage might also determine leakage. In order to eliminate the effects of reaction time and DNA concentration, we usually used a 24-h observation period and hairpin species concentration of 5 μM above. This DNA concentration was at least one or two order of magnitude higher than those typically used in enzyme-free nucleic acid dynamical systems (9). In addition, in order to further verify the effect of reaction time, three hairpin species were investigated further without initiators in the reaction buffer for 48 and 72 h at 37°C. Native polyacrylamide gel electrophoresis analysis showed that each hairpin species had not significant change in leakage within 48 h or even 72 h (Supplementary Figure S11A–F).

**Figure 5.**
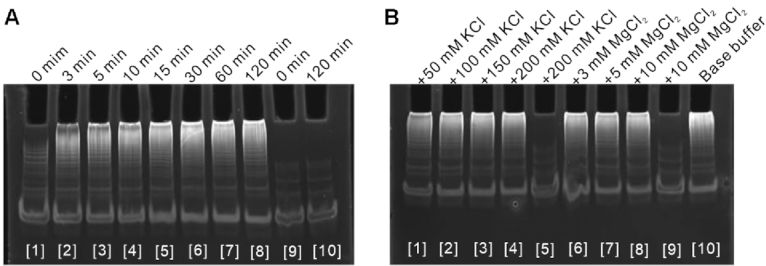
Effects of component concentration and reaction condition on DNA assembly behavior. (**A**) Native polyacrylamide gel electrophoresis assay showing the time-dependent assembly efficiency for H1b-17, H2b-17 and initiator in the optimized buffer. Lanes 1–8: assembly efficiency for H1b-17, H2b-17 and initiator; Lanes 9–10: leakage evaluation for H1b-17 and H2b-17. (**B**) Native polyacrylamide gel electrophoresis assay showing the effect of ions on leakage. Lanes 1–4, 6–8 and 10: assembly efficiency for H1b-17, H2b-17 and initiator; Lanes 5 and 9: leakage evaluation for H1b-17 and H2b-17. Base buffer represents the reaction buffer. The symbol + represents the addition of extra salt concentration.

Because the high salt concentration can hinder the migration rate in electrophoresis analysis, as mentioned above, we used an optimized buffer as the reaction buffer. This buffer contained a relatively low salt concentration. Native polyacrylamide gel electrophoresis analyzed the time-dependent assembly efficiency for H1b-17, H2b-17 and initiator at 37°C in the reaction buffer. The result showed that the assembly output increased quickly, whereas leakage did not occur over time (Figure 5A). In a real scenario, detection needs to work under different ionic conditions. Therefore, we further validated the robustness by adding ions to the reaction buffer. To evaluate the effect of KCl concentration, the reaction buffer was added 50, 100, 150 or 200 mM KCl. These buffers were tested in HCR of three hairpin species for 24 h at 37°C. Native polyacrylamide gel electrophoresis analysis showed that there was no significant difference in assembly efficiency for the same hairpin species and no leakage even at the highest KCl concentration (Figure 5B and Supplementary Figure S11G-H). In addition, we evaluated the effect of MgCl_2_ concentration by adding 3, 5 or 10 mM MgCl_2_ to the reaction buffer. Just like KCl, the similar result was obtained for MgCl_2_ (Figure 5B and Supplementary Figure S11G-H). These suggested that external factors have a limited impact on leakage and a key insight was the design of DNA hairpin that had an intrinsically higher energy barrier.

### Effect of initiator on DNA assembly behavior

**Figure 6.**
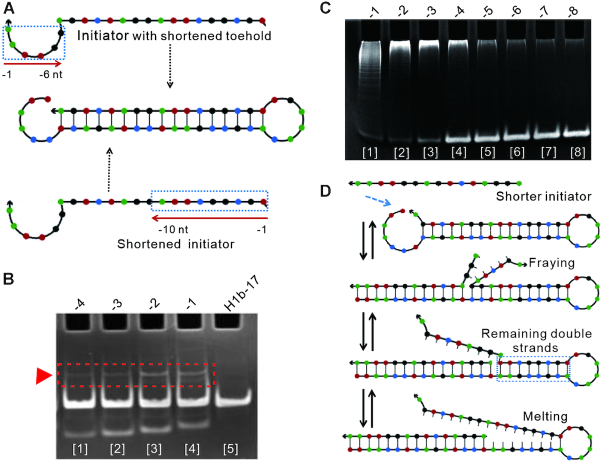
Effect of initiator on DNA assembly behavior. (**A**) Two possible kinds of initiators were listed. (**B** and **C**) Native polyacrylamide gel electrophoresis assay for evaluating the listed initiators in the reaction buffer at 37°C. (B) The triangle indicates the dimmers between H1b-17 with initiators. The values −4, −3, −2 and −1 represent the number of truncated nucleotides for the toehold, and they correspond to T-1, T-2, T-3 and T-4, respectively. (C) The hybridization products were showed. The values from −1 to −8 represent the number of truncated nucleotides for initiators, and they correspond to initiators from T5 to T12. (**D**) Principle of the effect of initiator length on leakage. Remaining double strand was melted at the branch point.

In a real analytical scenario, there will be many highly similar sequences, such as miRNAs, that perhaps cause HCR with the similar target and reduce the specificity of the analysis. Given this situation, we evaluated the impact of two possible kinds of initiators on HCR in the reaction buffer at 37°C by native polyacrylamide gel electrophoresis (Figure 6A and Supplementary Table S8).

First, initiators with shortened toeholds were incubated with H1b-17 separately for 10 min. The results showed that the dimers were produced in each system (Figure 6B). In this way, if there was a difference in the toehold binding area for the detection of targets, the reaction could still occur, reducing the specificity of the analysis.

Next, shortened initiators with a constant toehold length were incubated with H1b-17 and H2b-17 separately for 2 h. The results showed that the hybridization products were absent when the initiator truncated by about 6 nt (Figure 6C). As a parallel test, SERS was used for readouts (Supplementary Figure S12A). To further confirm this finding, other three hairpin species were also tested, and the same results were showed (Supplementary Table S8 and Figure S12B−E). We speculated that the reason might be also transient melting of the remaining double-strand DNA within 5–7 bp length after the initiator performed the displacement (Figure 6D). If the remaining double-strand DNA was longer, the reaction would not occur. Therefore, we thought it might be instructive to distinguish the highly similar sequences by HCR. In other words, if there is a mutation site at 5–7 bases far away from the end of the sequence, it may be unable to trigger the HCR. In this way, similar sequences may be distinguished.

### Optimized design of leak-resistant HCR for specific signal amplification of miRNA

**Figure 7.**
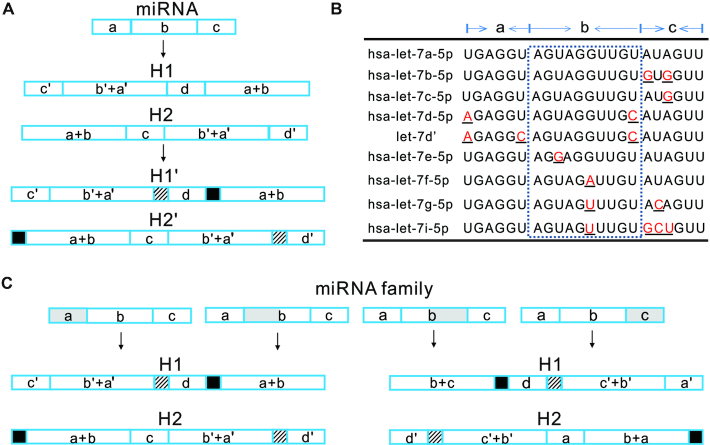
Optimized design of leak-resistant HCR for specific signal amplification of miRNA. (**A**) Design framework of hairpins for single miRNA. (**B**) The let-7 family and analog. miRNA was *subdivided* into into three parts. (**C**) Design framework of hairpins for highly similar miRNA. a, b and c represent three parts of miRNA, and d represents the sequestered loop. a’, b’, c’ and d’ represent complementary sequences of a, b, c and d, respectively. Gray squares represent the presence of different bases. The squares with stripes and the black ones represent the added bases, and they complement each other.

The basic framework for hairpin design was described in method section. To improve specificity for signal amplification of miRNA, we proposed that it was possible to resist leakage through a testing benchmark of −16.0 kcal mol^−1^ as mentioned. Therefore, if the free energy is too high, some bases need to be added to enhance the stability of the stem and avoid leakage. In addition, miRNA was *subdivided* into into three parts. About six bases at each end was respectively named the region a and c, and the remaining was region b. On these bases, the strategy was proposed to design hairpins for miRNA (Figure 7A).

Because many members within the same miRNA family share similar sequences such as the let-7 family, they may be able to trigger the same HCR and not be amplified specifically (Figure 7B). let-7d’ is also designed to be an analog of has-let-7d-5p (Figure 7B). In this regard, three strategies were further proposed to design hairpins below (Figure 7C).

- If the region a is different, c-complementary sequence is used as an exposed toehold; for instance, hsa-let-7d-5p and let-7d’.
- In contrast, if the region c is different, the a-complementary sequence is used as an exposed toehold; for instance, hsa-let-7b-5p and hsa-let-7c-5p.
- If the region b is different, choose a-complementary or c-complementary sequence as an exposed toehold based on which is farther from the difference site in region b, but did not have to; for instance, hsa-let-7a-5p, hsa-let-7e-5p and hsa-let-7f-5p.

As mentioned above, if there is a mutation site at 6–7 bases far away from the end of the sequence, it may be unable to trigger the HCR. To distinguish similar sequences, besides choosing the right toehold and regulating free energy, additional bases might also be required to prevent from being deceived.

### Specific signal amplification of miRNA

#### Signal amplification of hsa-miR-21-5p from exosomes

We used hsa-miR-21-5p as a target for illustrative purposes only. The designed hairpins based on the basic framework would lead to leakage, but leakage was avoided by lengthening one base pair under the guidance of free energy (Figure 2E and Supplementary Table S4). Here, HR1-17C and HR2-17C were used for amplification.

**Figure 8.**
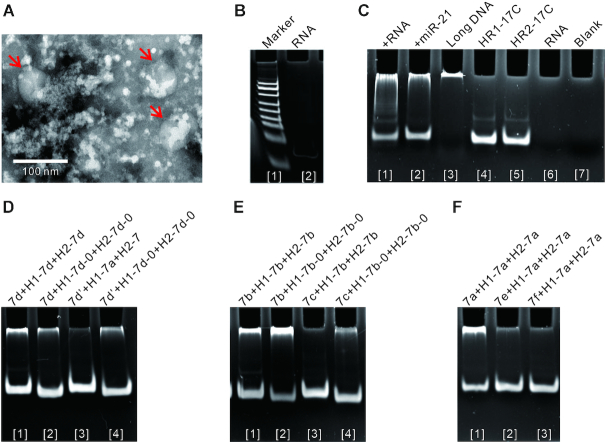
Specific signal amplification of miRNA. (**A**) Transmission electron microscope images of exosomes. The arrows indicate exosomes. (**B**–**F**) Native polyacrylamide gel electrophoresis assay showing leakage. (B) Total RNA from urinary exosomes. (C) Signal amplification of hsa-miR-21-5p. Lane 1: +RNA represents that total RNA as initiator was added in the system of HR1-17C and HR2-17C; lane 2: +miR-21 represents that hsa-miR-21-5p as the positive control was added in the system of HR1-17C and HR2-17C; lane 3: pET-28a was used as the positive control; lane 4: the monomer of HR1-17C, lane 5: the monomer of HR2-17C; lane 6: only the same concentration of RNA as lane 1; and lane 7: blank. (D) hsa-let-7d-5p was distinguished from let-7d’. c-complementary sequence was used as an exposed toehold. 7d represents hsa-let-7d-5p, and 7d’ represents let-7d’. (E) hsa-let-7b-5p was distinguished from hsa-let-7c-5p. a-complementary sequence was used as an exposed toehold. 7b represents hsa-let-7b-5p, and 7c represents hsa-let-7c-5p. (F) hsa-let-7a-5p was distinguished from hsa-let-7e-5p and hsa-let-7f-5p. c-complementary sequence was used as an exposed toehold. 7a represents hsa-let-7a-5p, 7e represents hsa-let-7e-5p and 7f represents hsa-let-7f-5p.

Next, exosomes were separated, and their size was distributed around ∼100 nm (Figures 8A and Supplementary S13A). SDS polyacrylamide gel electrophoresis analysis showed an obvious band at ∼60 kDa (Supplementary Figure S13B), which might include the exosome-specific external CD63 protein (41). Furthermore, RNA was also extracted (Figure 7B) and incubated with HR1-17C and HR2-17C in the reaction buffer for 2 h at 37°C. Native polyacrylamide gel electrophoresis and SERS were performed to detect the hybridization products (Figure 7C and Supplementary Figure S14A). In addition, the reliability of urine samples from nine patients was also performed (Supplementary Figure S14B). The results confirmed that the design method of leak-resistant HCR was valid for signal amplification of miRNA from biological samples.

#### *Distinguishing of hsa-let-7 family* members

We used *hsa-let-7 family* and analog as targets for illustrative purposes only (Figure 7B). Following above rules, DNA hairpins were designed to assess the feasibility of *distinguishing miRNA* in the reaction buffer for 2 h at 37°C.

Because hsa-let-7d-5p was different from let-7d’ in region a, c-complementary sequence was used as an exposed toehold. Then, H1-7d-0 and H2-7d-0 were designed based on the basic framework (Supplementary Table S9). Although they fit the testing benchmark of free energy, the hairpins still need to add extra bases in order to prevent from being deceived. Further, H1-7d and H2-7d were designed. Compared with H1-7d-0 and H2-7d-0, H1-7d and H2-7d had the ability to distinguish hsa-let-7d-5p from let-7d’ (Figure 8D). Similarly, after choosing a-complementary sequence as an exposed toehold, H1-7b and H2-7b were further designed and showed the ability to distinguish hsa-let-7b-5p from hsa-let-7c-5p (Figure 8E).

Hsa-let-7a-5p was different from both hsa-let-7e-5p and hsa-let-7f-5p in region b. Because the region c is relatively far from the different base, c-complementary sequence was chosen as an exposed toehold. H1-7a and H2-7a were further designed and showed the ability to distinguish hsa-let-7a-5p from hsa-let-7e-5p and hsa-let-7f-5p (Figure 8F).

In addition to the above, more distinctions among members were implemented (Supplementary Figure S14C-D and Table S9). The results confirmed that the design method of leak-resistant HCR was valid for the specific analysis of miRNA family.

## DISCUSSION

A number of leak reduction methods have been proposed for DNA strand displacement: using highly purified DNA (9), minimizing sequence symmetry (4), closing the ends of the fraying region with C/G (16) or DNA clamp (4), introducing mismatch modifications at the fraying region (14), incorporating LNA nucleotides into DNA hairpin (42), engineering split proximity circuit (22), constructing a parallel shadow circuit (20) and using multiarm junction structures (21). To sum up these methods, they are inseparable from one purpose of creating high energy barrier to block undesired toehold strand displacement by either sequence design or physically separating, especially zero toehold event via thermal fluctuations at the duplex ends or nicks. In fact, our work was also around this purpose.

The stability of DNA is determined by nearest neighbor stacking and hydrogen bonding free energies (43). A fundamental question is how much free energy can break a section or the whole of a DNA double helix (23). Regarding this issue, a definitive statement cannot be provided. Rather, it seems to be impossible to quantify the motility without an adequate theoretical treatment of DNA breathing fluctuations (44). DNA melting is the process of separation of DNA *double strands* into two single strands. The temperature at which half of the DNA molecule is denatured is termed as the melting temperature of the DNA, Tm (45). From the definition, *T*_m_ cannot determine a specific state of DNA though it is an important parameter. To compare the relative stability of DNA hairpin species, we calculated the free energy of the secondary structure by NUPACK, which was defined in 1 M Na^+^ as a testing benchmark (46). These reference values may change by depending on ion parameters and software updates. However, they can be corrected according to the sequence correspondingly.

For two DNA hairpins in HCR, the most obvious feature is that the input domain is sequestered in hairpin. The question is how leakage occurs without initiator. Because the details of the biophysical mechanism and intermediate process have remained elusive (40,47), two internal paths were hypothesized in general. Based on the hypothesis of entry to active breathing sites next to loop, we considered three possibilities for the initial status: (i) the exposed toehold easily crosses with the loop sequestered as a toehold, (ii) the exposed toehold accidentally crosses with the loop sequestered as a toehold, or (iii) binding never occurs, but the DNA frays directly at active breathing sites next to the loop. In any case, the initial leakage must access active breathing sites in the absence of DNA melting. From the point of view of dynamics and biophysics, the hypothesis is further denied.

To support the hypothesis of transient DNA melting, we investigated whether leakage depends on the stem sequences and free energy in solvent conditions. But we didn’t answer how it happened below the melting temperature. We speculated that two identical hairpins may be able to fray by thermal fluctuations at the open end of hairpin stem, then from the four-way branch migration. Depending on the stem sequences and free energy, the branched chain may be inclined to melt transiently at some point and expose the hairpin loop, resulting in leakage. In addition, because the hairpin structure is usually formed by annealing, two identical hairpins may hybridize each other and form a four-branch migration structure. More details are needed.

In this work, we find out that the shorter stem with the higher free energy was inclined to cause leakage, whereas the longer stem was the opposite. From all the data, we proposed that it was possible to resist leakage through a testing benchmark of −16.0 kcal mol^−1^. To affirm the generality of this discovery, we used as many sequences as possible. But after all, DNA sequences are endless, and it is impossible to verify them one by one. In organisms, miRNA sequences are abundant and need to be amplified by HCR. That's why we chose a lot of miRNAs in our tests.

Although it is known that the strand displacement is thermodynamically driven forward and the kinetics can vary a million-fold depending on the length of the toehold (1), little is known how to generalize the results. By evaluating the initiator, we found that shortened initiators with a constant toehold length were more effective in controlling the reaction. Based on our findings, leak-resistance HCR was extended to distinguish miRNA. It was tentatively suggested that the remaining double chains could melt within 6 or 7 bp after the initiator performed the displacement. According to our hypothesis and experimental dates, such as HR1-15 and HR1-16, hairpins with stem of more than 10 bp can even melt to cause leakage. Thus, they seem inconsistent. We speculated there are also dynamics reasons besides thermodynamics. Because shortened initiators cannot completely displace the stem, they move back and forth at the branch point as an unbiased random walk, and the stem cannot be dissociated. Of course, this value may also change with the reaction condition, and we are also trying to establish a quantitative criterion. If more details are known on the biophysics and kinetics, this may be of great significance for specificity analysis.

At last, HCR promotes the formation of longer DNA chains from short hairpin monomers, and it is difficult to devise a simple readout strategy based on the change in molecular weight, except by using gel electrophoresis, which is not practical for actual detection applications. Optimized methods based SERS will be reported in the future.

## Supplementary Material

gkaa016_Supplemental_File
