# Supplementary material for: Elucidation of leak-resistance DNA hybridization chain reaction with universality and extensibility
Source: Nucleic Acids Res. 2020 Feb 5;48(5):2220–31. doi: 10.1093/nar/gkaa016 (PMC7049695; doi:10.1093/nar/gkaa016)
Supplement: gkaa016_Supplemental_File [file gkaa016_supplemental_file.pdf]

SUPPLEMENTARY DATA

Elucidation of leak-resistance DNA hybridization chain reaction with universality and extensibility

Shaofei Li[1,2,4], Pan Li[1], Meihong Ge[1,4], Hongzhi Wang[1,3], Yizhuang Cheng[1,4], Gan Li[5], Qiang Huang[5], Huan He[1], Chentai Cao[1,4], Dongyue Lin[1,4], Liangbao Yang[1,3,*]

[1] Center of Medical Physics and Technology, Hefei Institutes of Physical Science, Chinese Academy of Sciences, Hefei, Anhui 230031, China
[2] School of Life Science, Anhui University, Hefei, Anhui 230601, China
[3] Cancer Hospital, Chinese Academy of Sciences, Hefei, Anhui 230031, China
[4] Department of Chemistry, University of Science and Technology of China, Hefei, Anhui 230026, China
[5] State Key Laboratory of Genetic Engineering, School of Life Sciences, Fudan University, Shanghai 200438, China

* To whom correspondence should be addressed. Email: lbyang@iim.ac.

Contents

List of Supplementary Texts:

Text S1. Molecular dynamics simulation

List of Supplementary Figures:

Figure S1. Native polyacrylamide gel electrophoresis and SERS analysis method

Figure S2. Effect of stem length on DNA assembly behavior

Figure S3. Effect of reversed stem length on DNA assembly behavior

Figure S4. Leakage of DNA hairpins based on other sequences

Figure S5. Effect of free energy on DNA assembly behavior

Figure S6. Leakage of hairpin sequences at different temperature

Figure S7. Linear fit of free energy versus length of the sequences at different temperature

Figure S8. Effects of toehold and loop on DNA assembly behavior

Figure S9. Stability analysis of enlarged loops

Figure S10. Stability analysis of other enlarged loops

Figure S11. Effects of DNA concentration and reaction condition on DNA assembly behavior

Figure S12. Effect of initiator on DNA assembly behavior

Figure S13. Isolation and characterization of exosomes

Figure S14. Specific signal amplification of miRNA

List of Supplementary Tables:

Table S1. Evolution of the hairpin based on Dirks and Pierce's

Table S2. Evolution of the hairpin based on reversed stem

Table S3. Evolution of the other hairpin based on Dirks and Pierce's

Table S4. Evolution of the other DNA Hairpin sequences

Table S5. Evolution of the hairpin based on changed toehold

Table S6. Modified sequences in stem region by mutation and substitution

Table S7. DNA sequences used in effect of toehold and loop length on DNA assembly behavior

Table S8. DNA sequences used in effect of initiator on HCR

Table S9. DNA Fairpin for detection of miRNA family

Supplementary Text S1. Molecular dynamics simulation

Molecular dynamics was used to simulate the states of DNA in a solvent environment. First, a 3D atomic model of DNA was built with the web server 3D-Nus. Then, in each simulation system, a water cube with a DNA model in its centre was established, and the distance from the cube edges to the model surface was kept to a minimum of 12 Å. To achieve an ionic concentration of 0.15 M, the appropriate proportion of water was replaced with Na^+ and Cl^- ions, with extra Na^+ ions included for charge neutralization.

All simulations were performed with the program Gromacs-5.0.7, and periodic conditions were applied. The pressure was balanced with standard atmospheric pressure by the Berendsen method, and the temperature was maintained at 300 K by velocity rescaling. The particle mesh Ewald (PME) method was used to calculate the electrostatic interactions, and the cut-off radius for both electrostatic and van der Waals interactions was set to 14 Å. All bonds involved in the system were constrained with the LINCS algorithm, and a time step of 2.0 fs was used.

The force field was selected scrupulously from a series, and eventually Parmbsc1 was selected for use in all the simulations, since it performed perfectly in the DNA atomistic simulations(1). The general form of the molecular force field is:

$$\begin{aligned} \mathrm{U} &= U_b + U_\theta + U_\phi + U_\chi + U_{vdW} + U_{el} \\ &= \frac{1}{2}\sum_i k_b\,(r_i - r_{i0})^2 + \frac{1}{2}\sum_i k_\theta\,(\theta_i - \theta_{i0})^2 + \frac{1}{2}\sum_i V_n(1+\cos(n\phi - \delta)) \\ &+ \frac{1}{2}\sum_i k_\chi\,\chi^2 + \sum_i \sum_{j=i+1} 4\varepsilon_{ij}\left[(\frac{\sigma_{ij}}{r_{ij}})^{12} - (\frac{\sigma_{ij}}{r_{ij}})^6\right] + \sum_i \sum_{j=i+1} \frac{q_i q_j}{4\pi\varepsilon_0\varepsilon_r r_{ij}} \end{aligned}$$

where r_{i0}, θ_{i0} stands for standard values of bond length and angle; k_b, k_θ stands for elastic coefficient of harmonic potential energy; n stands for the rotation period of the dihedral angle; δ stands for the phase; $V_n k_\chi$ stands for the height of the barrier; ε_{ij} stands for the depth of the potential well of the interaction between atom i and j; σ_{ij} stands for the distance between two atoms when the potential energy of van der Waals is minimum; $q_i q_j$ stands for the charge of atom i and j respectively; and r_{ij} stands for the distance between atom i and j.

The parameterization processes of the force field Parmbsc1 uses a Monte Carlo method to avoid changes in other torsional parameters. It efficiently maintains the force field by fitting the QM-MM difference or residual energy to a Fourier series in the third order(2). It can be represented as follows:

$$E_{dih} = E_{QM} - E_{ffbsc0(x=0)} \qquad \text{(I)}$$

$$E_{dih} = \sum_{torsions} \sum_n^3 \frac{V_n}{2}[1 + \cos(n\varphi - \alpha)] \qquad \text{(II)}$$

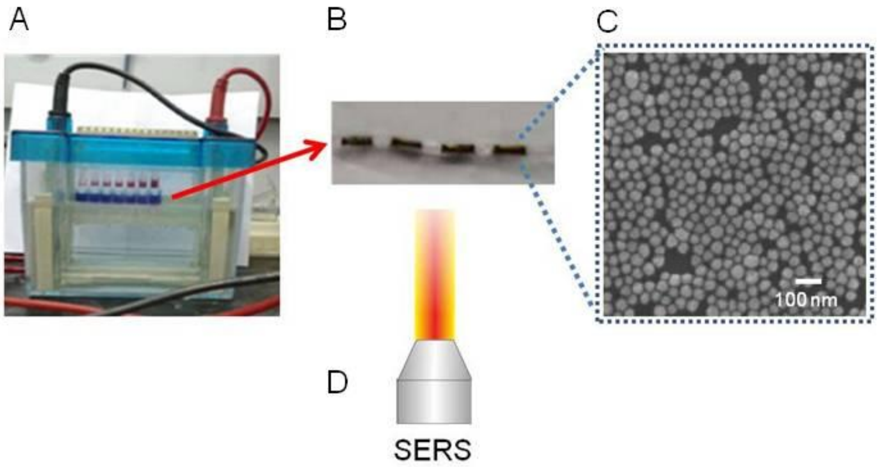

Supplementary Figure S1. Native polyacrylamide gel electrophoresis and SERS analysis method. (A) Native polyacrylamide gel electrophoresis with the coated gold nanoparticles. (B) Electrophoretic gel surface in the slot. (C) Nanoparticles characterized by transmission electron microscope. (D) The diagram of SERS detection.

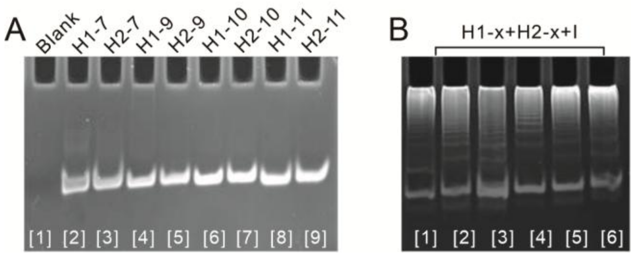

Supplementary Figure S2. Effect of stem length on DNA assembly behavior. (A) and (B) Native polyacrylamide gel electrophoresis analysis as control. (A) The hairpin monomers were analyzed, and no leakage products were produced in the gel loading well. (B) Stem lengths of 13 to 20 bp were tested in response to initiator. Hybridization products were blocked in the gel loading well. x represents the values 13, 15 and 17–20. I represents the initiator sequence.

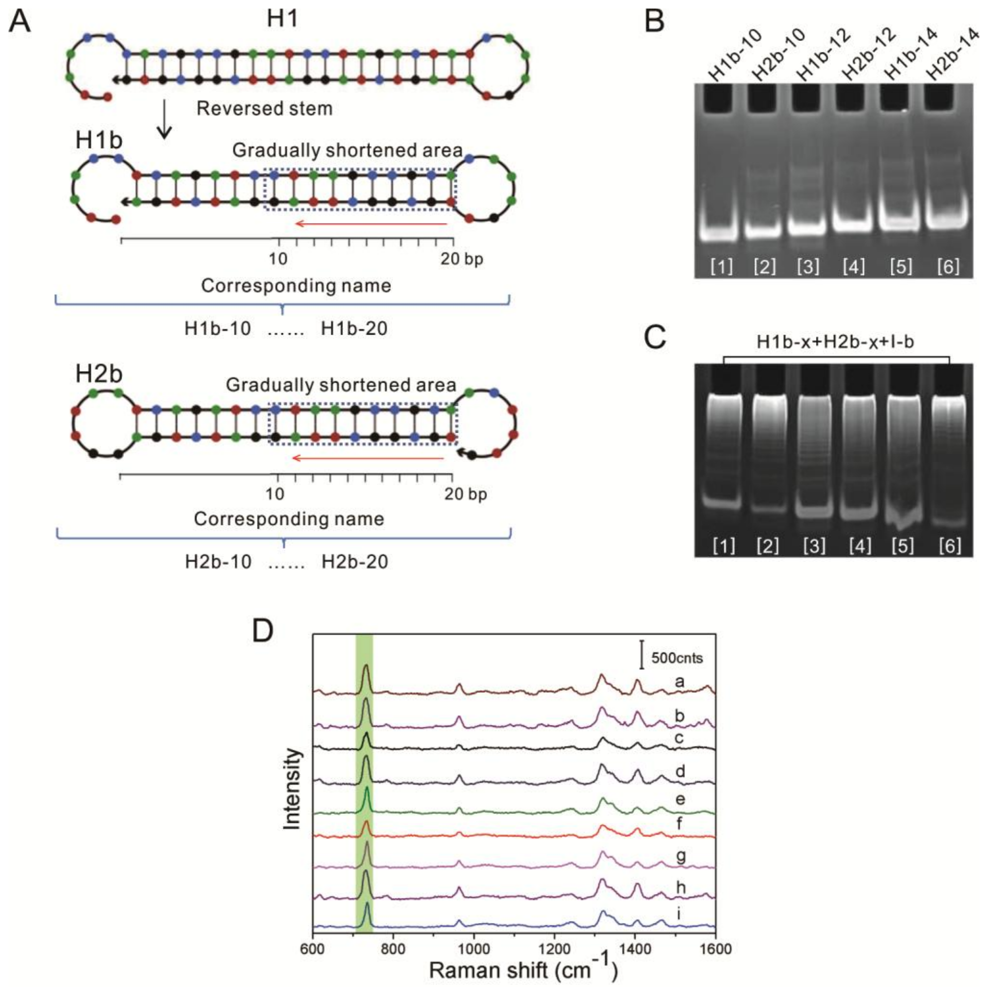

Supplementary Figure S3. Effect of reversed stem length on DNA assembly behavior. (A) Evolution of the hairpin design. The reversed stem length was changed. bp represents the base pairs.(B) Native polyacrylamide gel electrophoresis analysis for the monomer of DNA hairpin. (C) Native polyacrylamide gel electrophoresis analysis. Normal hybridization products were blocked in the gel loading well. x represents the values 15-20, and I-b represents the initiator sequence. (D) SERS analysis for leakage in the gel loading well. The green band indicates the marker of adenine at approximately 732 cm^{-1}. Spectra a-c correspond to lanes 1-3 in Figure 2D respectively, and d-i correspond to lanes 1-6 in Figure 2E respectively.

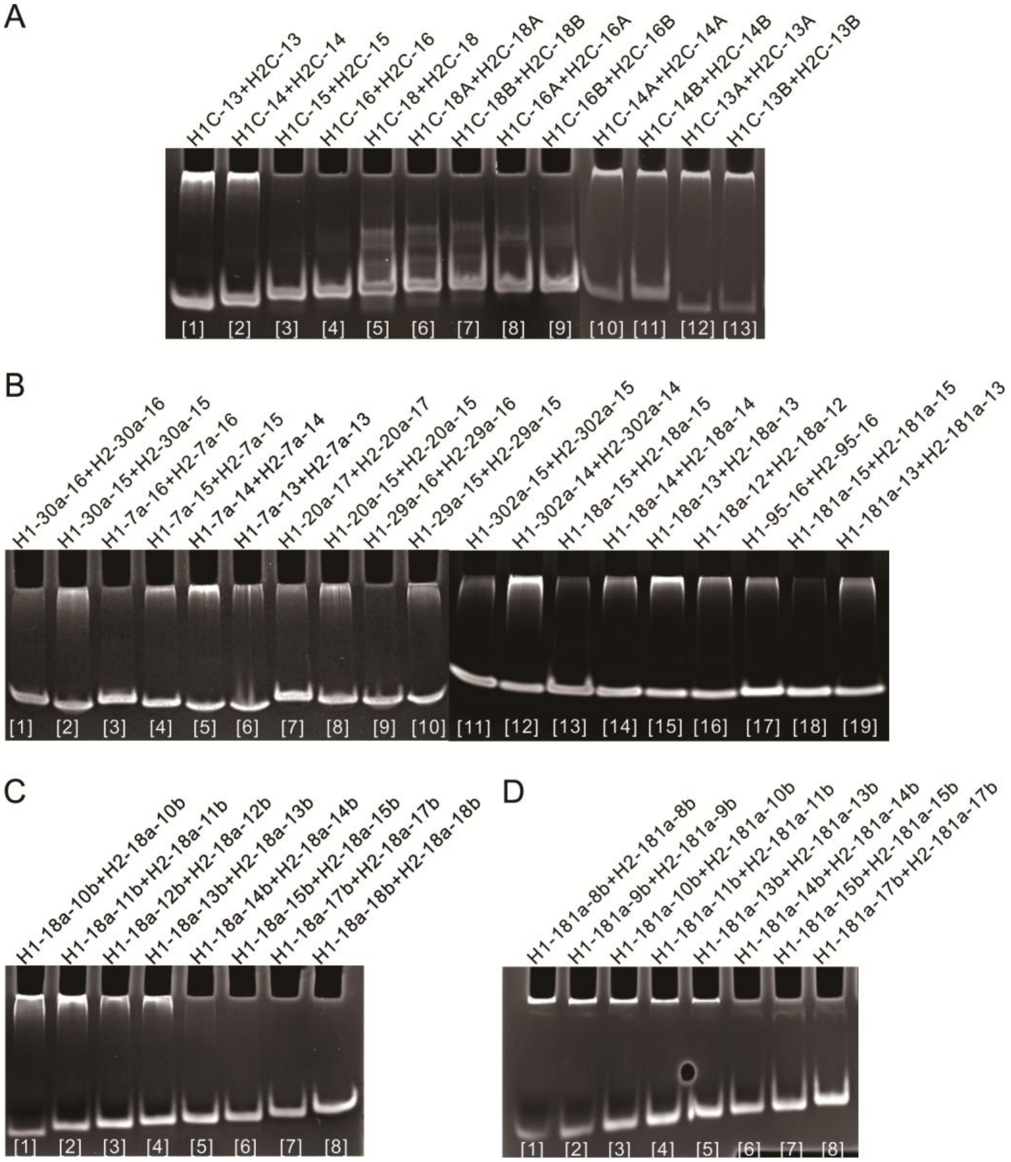

Supplementary Figure S4. Leakage of DNA hairpins based other sequences. (A), (B), (C) and (D) Native polyacrylamide gel electrophoresis analysis for leakage. Leakage products were blocked in the gel loading well. (A) The other DNA hairpins based on Dirks and Pierce's original design were also evolved by changing stem length and toehold base composition. H1C-13 to H1C-18 had the same toehold. HC1-18A, HC1-16A, HC1-14A and HC1-13A had the same toehold. HC1-18B, HC1-16B, HC1-14B and HC1-13A had the same toehold. That leakage occurred in the stem lengths of 13 to 14 bp but not in the longer stem, and three toeholds did not show difference in leakage. (B) The other DNA Hairpin sequences based on miRNA were analyzed. (C) and (D) The DNA Hairpin sequences based on changed toehold were designed.

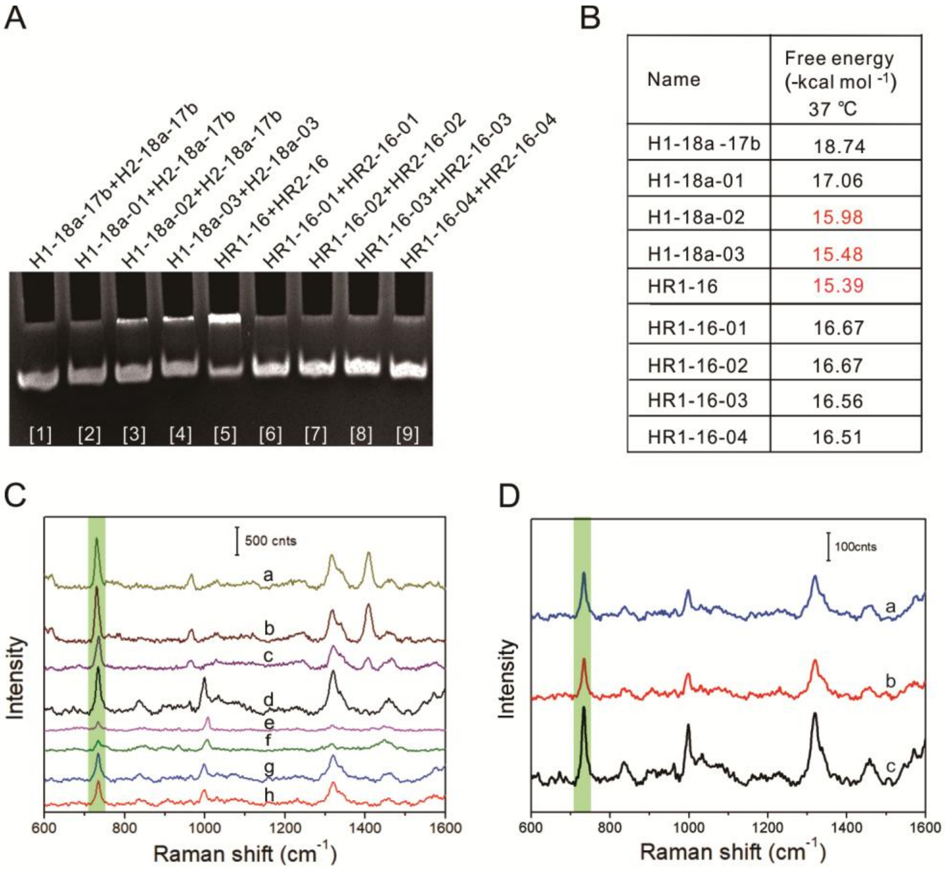

Name	Free energy (-kcal mol $^{-1}$) 37 ℃
H1-18a -17b	18.74
H1-18a-01	17.06
H1-18a-02	15.98
H1-18a-03	15.48
HR1-16	15.39
HR1-16-01	16.67
HR1-16-02	16.67
HR1-16-03	16.56
HR1-16-04	16.51

Supplementary Figure S5. Effect of free energy on DNA assembly behavior. (A) Native polyacrylamide gel electrophoresis assay. Sequences were modified in the stem region by mutation and substitution, and leakage occurred in sequences with higher free energy. Leakage products were blocked in the gel loading well. (B) The free energies of modified sequences. Leakage energies were marked in red. (C) and (D) SERS analysis for leakage in the gel loading well. The green band indicates the marker of adenine at approximately 732 cm^{-1}. (C) Spectra a-d correspond to lanes 6-9 in Figure 3B respectively. Spectra e-h correspond to lanes 3-4 and 6-9 in Figure 3C respectively. (D) Spectra a-c correspond to lanes 3-5 in panel A respectively.

A

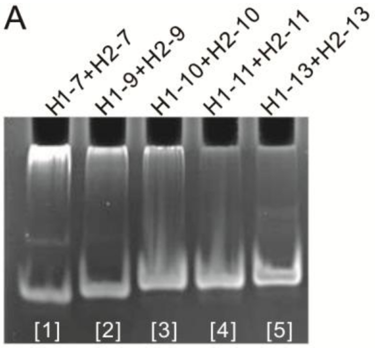

B

Name	Free energy (37 ℃)	Free energy (25 ℃)
H1-7	-8.53	-10.66
H1-9	-10.43	-13.03
H1-10	-11.51	-14.40
H1-11	-13.01	-16.15
H1-13	-15.93	-19.58

C

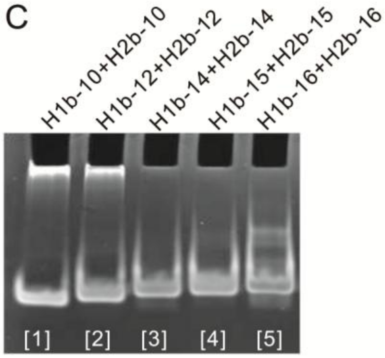

D

Name	Free energy (37 ℃)	Free energy (25 ℃)
H1b-10	-7.71	-10.13
H1b-12	-10.39	-13.39
H1b-14	-14.47	-17.97
H1b-15	-16.64	-20.50
H1b-16	-18.88	-23.00

E

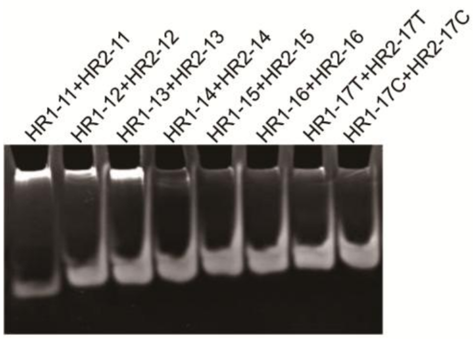

F

Name	Free energy (37 ℃)	Free energy (25 ℃)
HR1-17C	-17.23	-21.67
HR1-17T	-16.47	-20.91
HR1-16	-15.39	-19.53
HR1-15	-15.01	-18.94
HR1-14	-13.73	-17.59
HR1-13	-11.69	-15.10
HR1-12	-10.01	-13.10
HR1-11	-9.01	-11.83

Supplementary Figure S6. Leakage of hairpin sequences at different temperature. (A), (C) and (E) Native polyacrylamide gel electrophoresis analysis for leakage products at 25 °C. (B), (D) and (F) Comparison of free energy of sequences at different temperatures. The red value is the free energy of the leakage sequence. Leakage decreased accordingly as the temperature dropped.

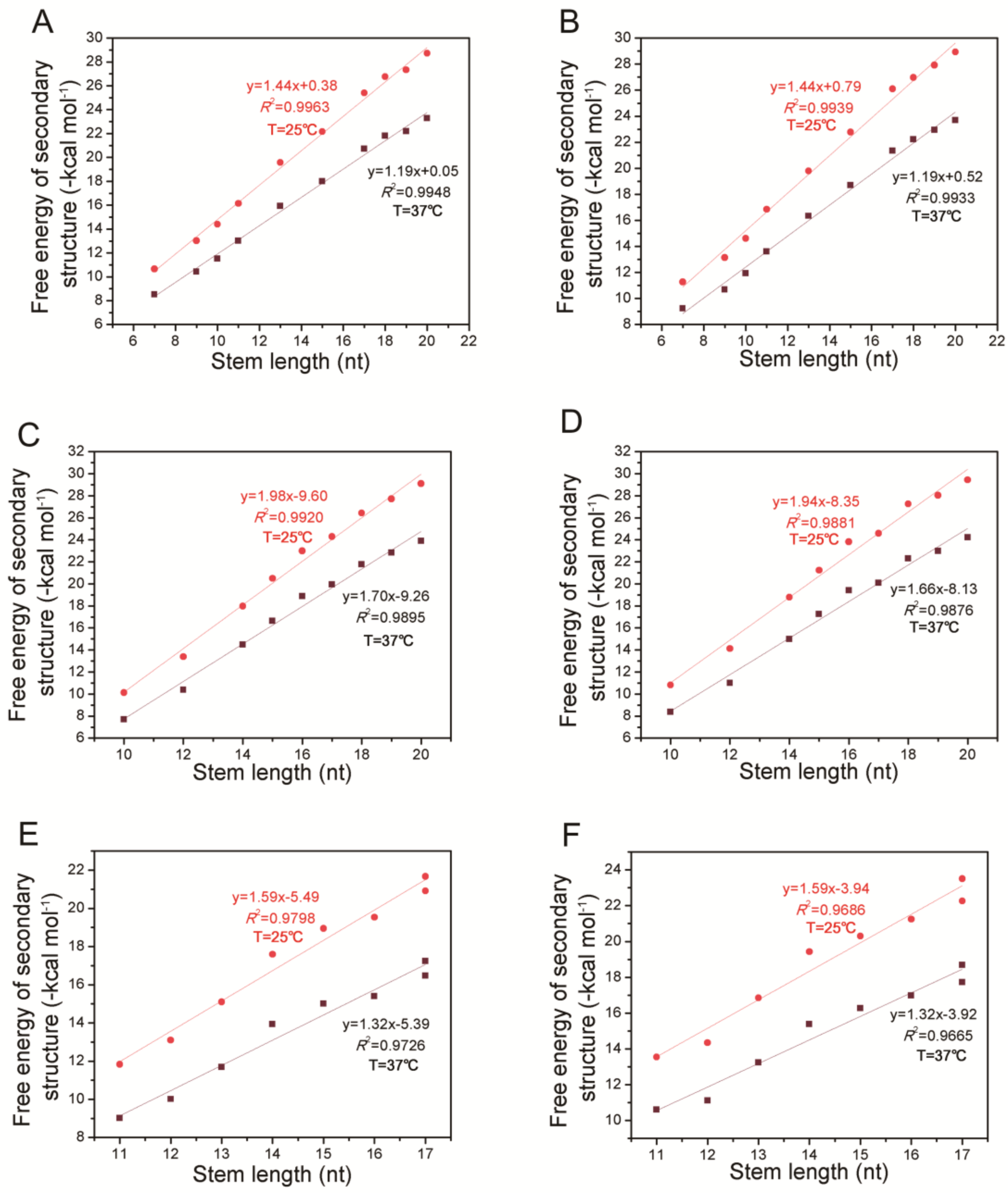

Supplementary Figure S7. Linear fit of free energy versus length of the sequences at different temperature. All of them showed a good linear relationship between the free energy of the secondary structure sequence and its length. (A) Sequences from H1-7 to H1-20. (B) Sequences from H2-7 to H2-20. (C) Sequences from H1-10b to H2-20b. (D) Sequences from H2-10b to H2-20b. (E) Sequences derived from HR1. (F) Sequences derived from and HR2.

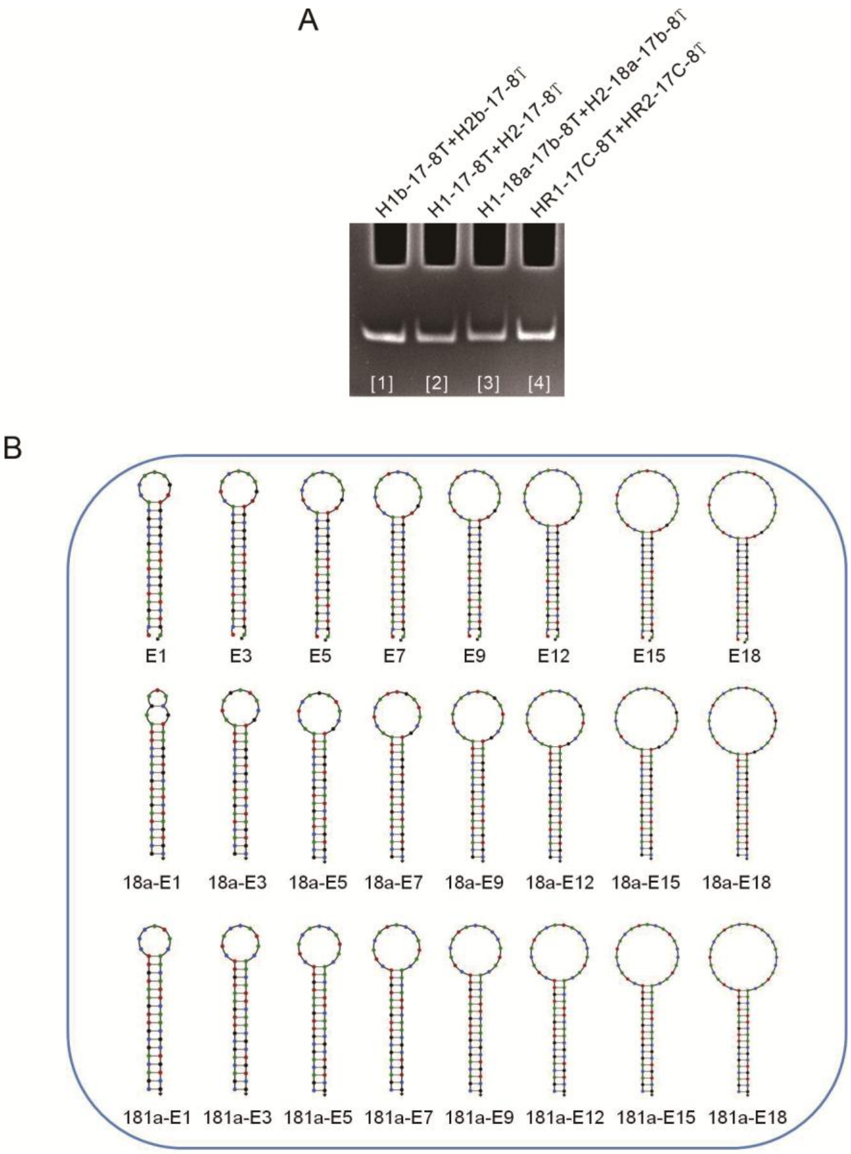

Supplementary Figure S8. Effects of toehold and loop on DNA assembly behavior. (A) Native polyacrylamide gel electrophoresis analysis. Four hairpin species with 8-nt exposed toehold and 8-nt loop were investigated, and no significant leakage occurred. (B) Minimum free energy structures of DNA hairpins with extended loops. Loops of three hairpin species based on H1b-17, H1-18a-17b and H1-181a-17b were enlarged by inserting bases. The enlarged loops did not form additional secondary structures.

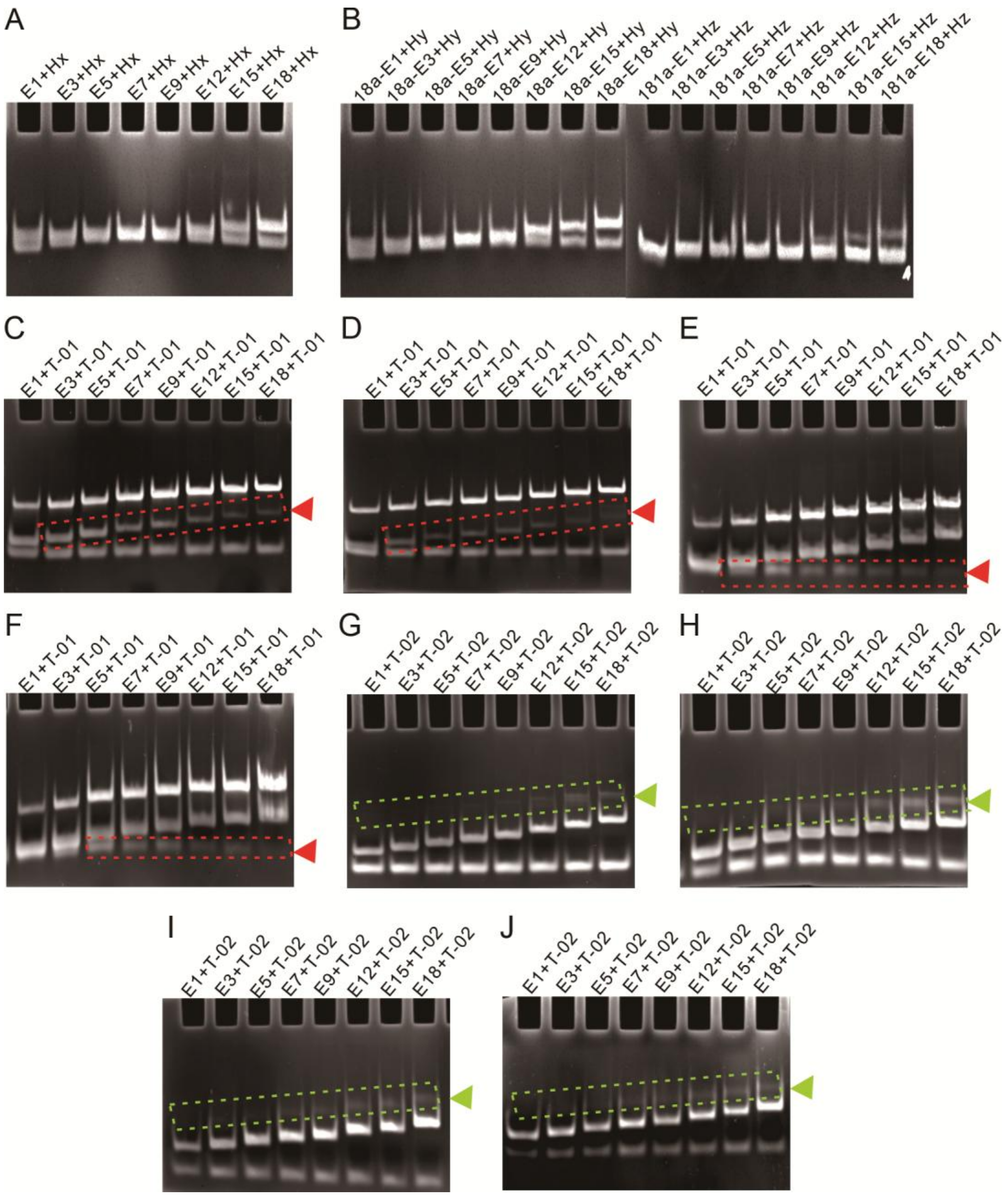

Supplementary Figure S9. Stability analysis of enlarged loops. (A) and (B) Native polyacrylamide gel electrophoresis assay for leakage with enlarged hairpin after incubation at 37 °C. No leakage products were blocked in the gel loading well. (A) For 24 h. Hx represents the hairpins H1b-17 and H2b-17. (B) For 48 h. Hy represents the hairpins H1-18a-17b and H2-18a-17b. Hz represents the hairpins H1-181a-17b and H2-181a-17b. (D)-(J) Native polyacrylamide gel electrophoresis assay for hybridization. T-01 or T-02 was incubated with corresponding hairpin. The red triangle indicated the consuming hairpins. The yellow-green triangle indicated the formed dimmers. (C) and (G) In the ratio of 3:2 for 2 h. (D) and (H) In the ratio of 3:2 for 12 h. (E) and (I) In the ratio of 2:3 for 2 h. (F) and (J) In the ratio of 2:3 for 12 h.

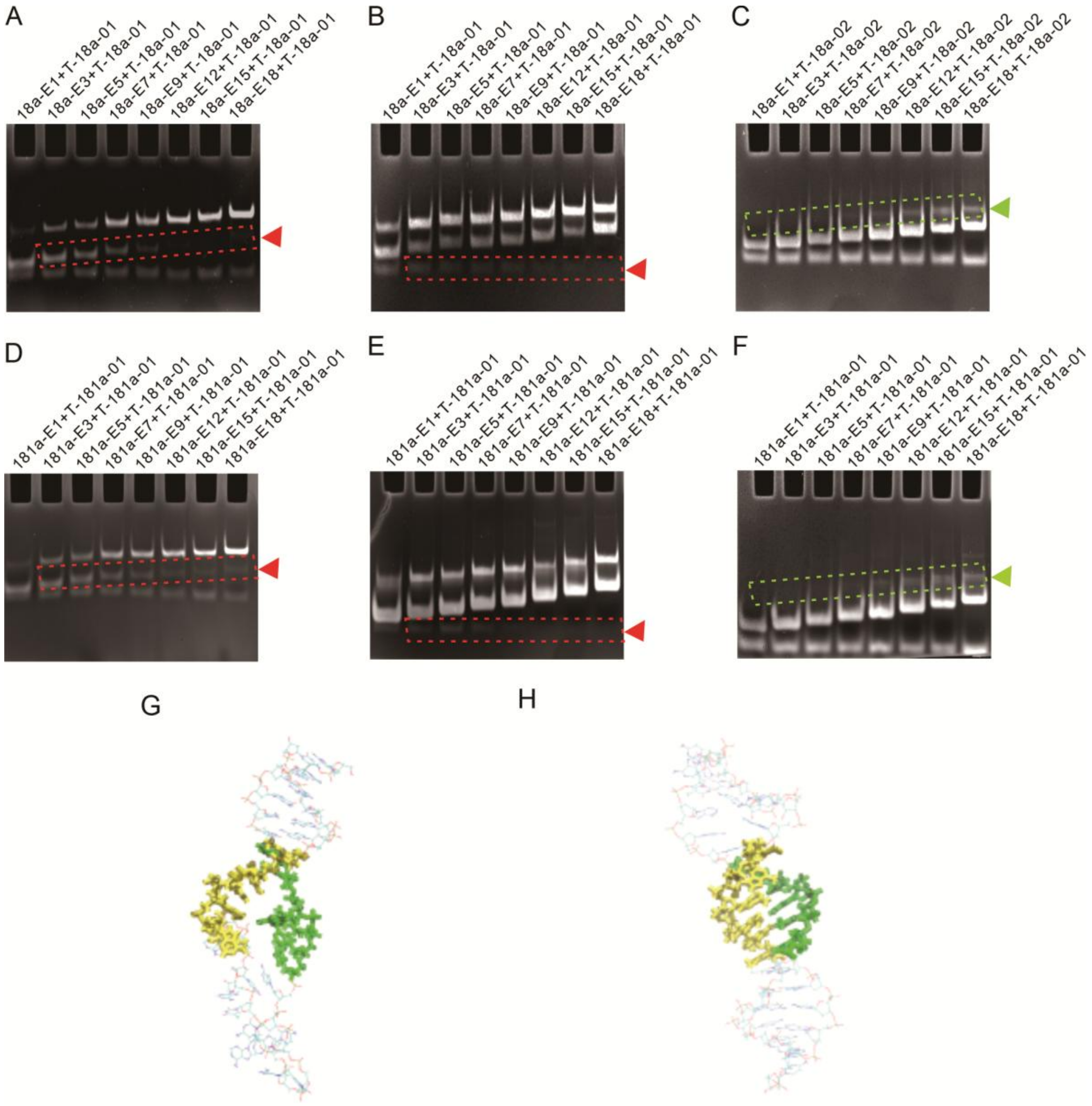

Supplementary Figure S10. Stability analysis of other enlarged loops. (A)-(F) Native polyacrylamide gel electrophoresis assay for hybridization. T-18a-01 or T-181a-01 was incubated with corresponding hairpin for 48 h. The consumption and products tended to occur in the systems of hairpins with longer loops. The red triangle indicated the consuming hairpins. The yellow-green triangle indicated the formed dimmers. (A) and (D) In the ratio of 3:2. (B) and (E) In the ratio of 2:3. (C) and (F) In the ratio of 3:2. (G) and (H) Molecular dynamics simulations for E18. The molecular conformation of the loop was shown in yellow. (G) Occasionally exposed state of the loop. (H) Restored state of the loop.

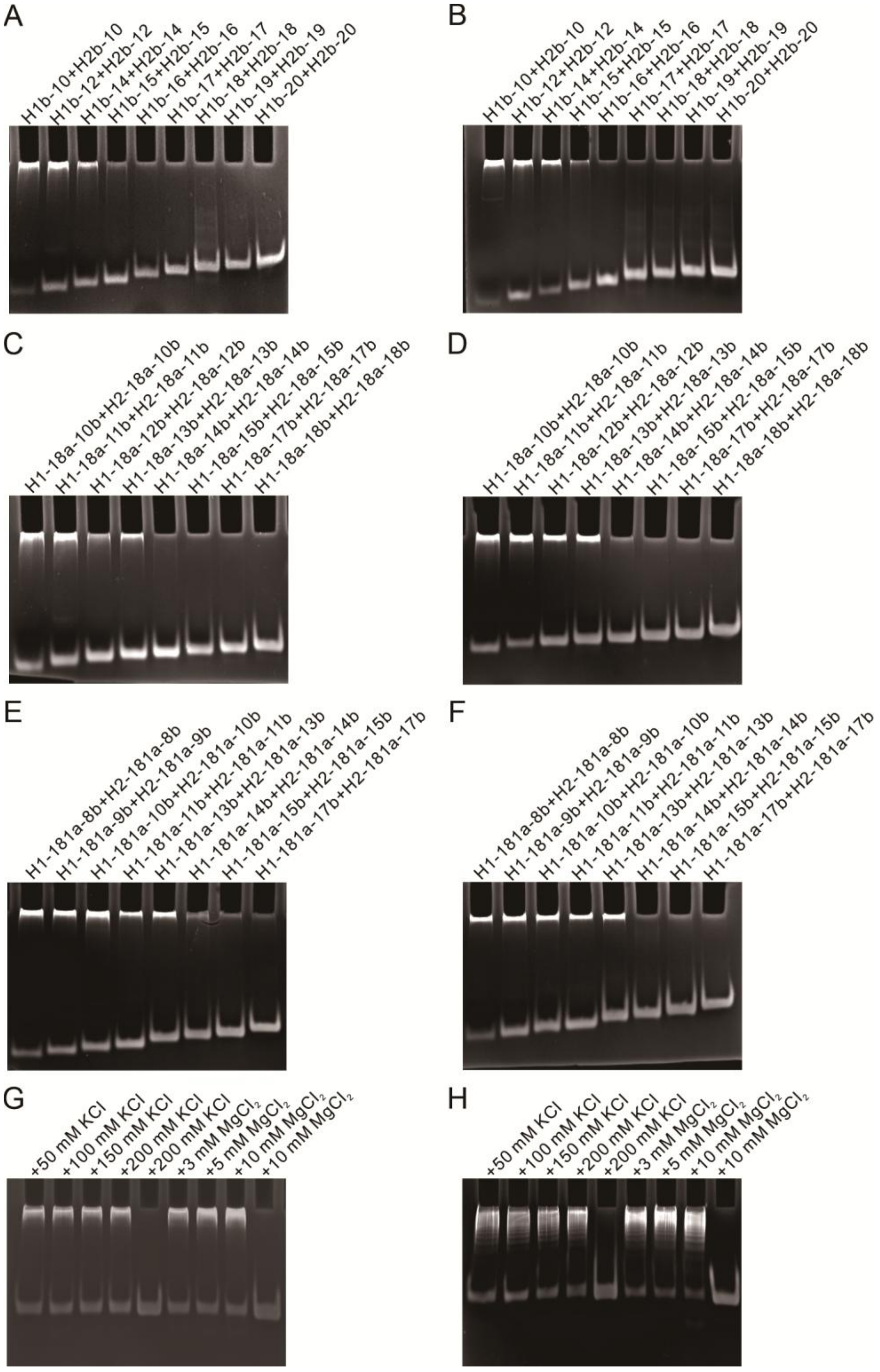

Supplementary Figure S11. Effects of DNA concentration and reaction condition on DNA assembly behavior. (A)-(F) Native polyacrylamide gel electrophoresis analysis for leakage. Three hairpin species were investigated without initiators in the reaction buffer at 37 °C, and no significant change in leakage. (A), (C) and (E) For 48 h. (B), (D) and (F) For 72 h. (G) and (H)

Native polyacrylamide gel electrophoresis assay showing the effect of ions on leakage. The symbol + represents the addition of extra salt concentration based on the reaction buffer. (G) Lanes 1–4 and 6-8: assembly efficiency for H1-18a-17b, H2-18a-17b and I-18a; Lanes 5 and 9: leakage evaluation for H1-18a-17b and H2-18a-17b. (H) Lanes 1–4 and 6-8: assembly efficiency for H1-181a-17b, H2-181a-17b and I-181a; Lanes 5 and 9: leakage evaluation for H1-181a-17b and H2-181a-17b.

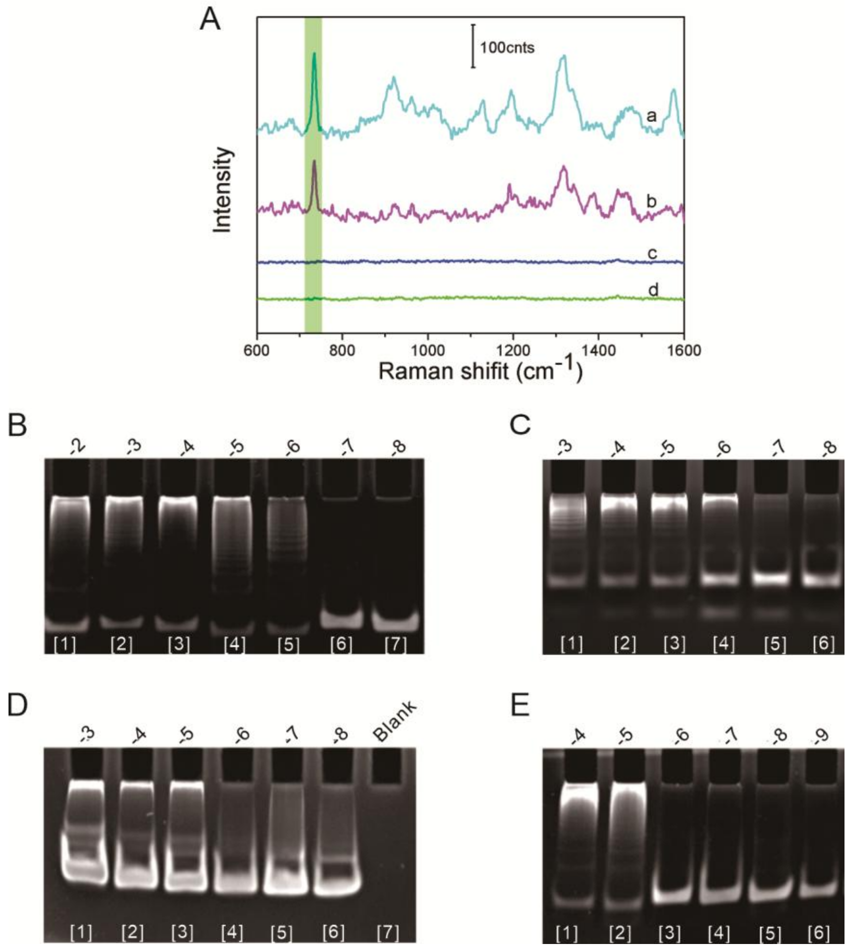

Supplementary Figure S12. Effect of initiator on DNA assembly behavior. (A) SERS showed the hybridization products. The green band indicated the marker of adenine at approximately 732 cm−1. Spectra a–d correspond to lanes 5–8 in Figure 6C respectively. (B)-(E) Native polyacrylamide gel electrophoresis assay for the hybridization products. The shortened initiators with a constant toehold length were incubated in the reaction buffer at 37 °C. The values from -2 to -9 represent the number of truncated nucleotides for initiators. The listed sequences are in Supplementary Table S8. (B) The hairpins are H1C-18 and H2C-18, and the initiators are from T-18-2 to T-18-8 respectively. They correspond to lanes 1–7 respectively. (C) The hairpins are H1-18a-17b and H2-18a-17b, and the initiators are from T-18a-3 to T-18a-8 respectively. They correspond to lanes 1–6 respectively. (D) The hairpins are H1d-20 and H2d-20, and the initiators are from T-20-3 to T-20-8. They correspond to lanes 1–6 respectively. (E) The hairpins are H1E-20 and H2E-20, and the initiators are from T-E20-4 to T-E20-9. They correspond to lanes 1–6 respectively.

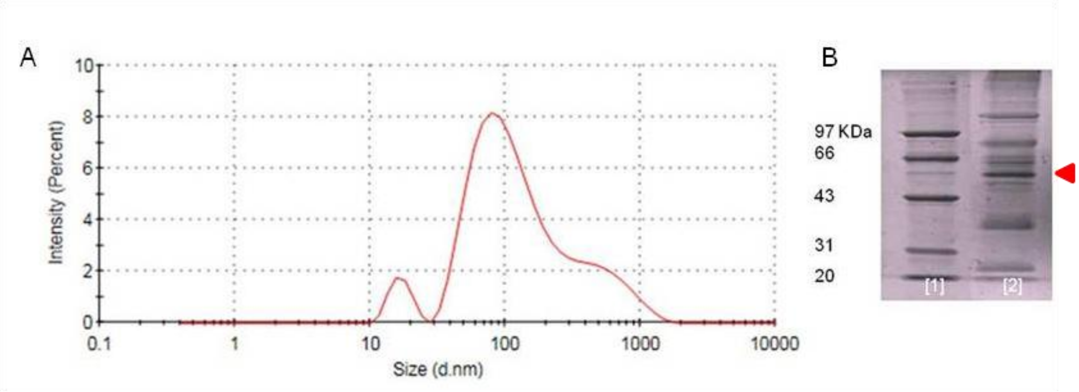

Supplementary Figure S13. Isolation and characterization of exosomes. (A) Size distribution of exosomes analysised by dynamic light scattering. The size was distributed around approximately 100 nm. (B) SDS polyacrylamide gel electrophoresis assay for the proteins of urinary exosomes. Lane 1: protein molecular weight standard; Lane 2: proteins of urinary exosomes. The red triangle indicates an obvious band at approximately 60 kDa, which might include the exosome-specific external CD63 protein.

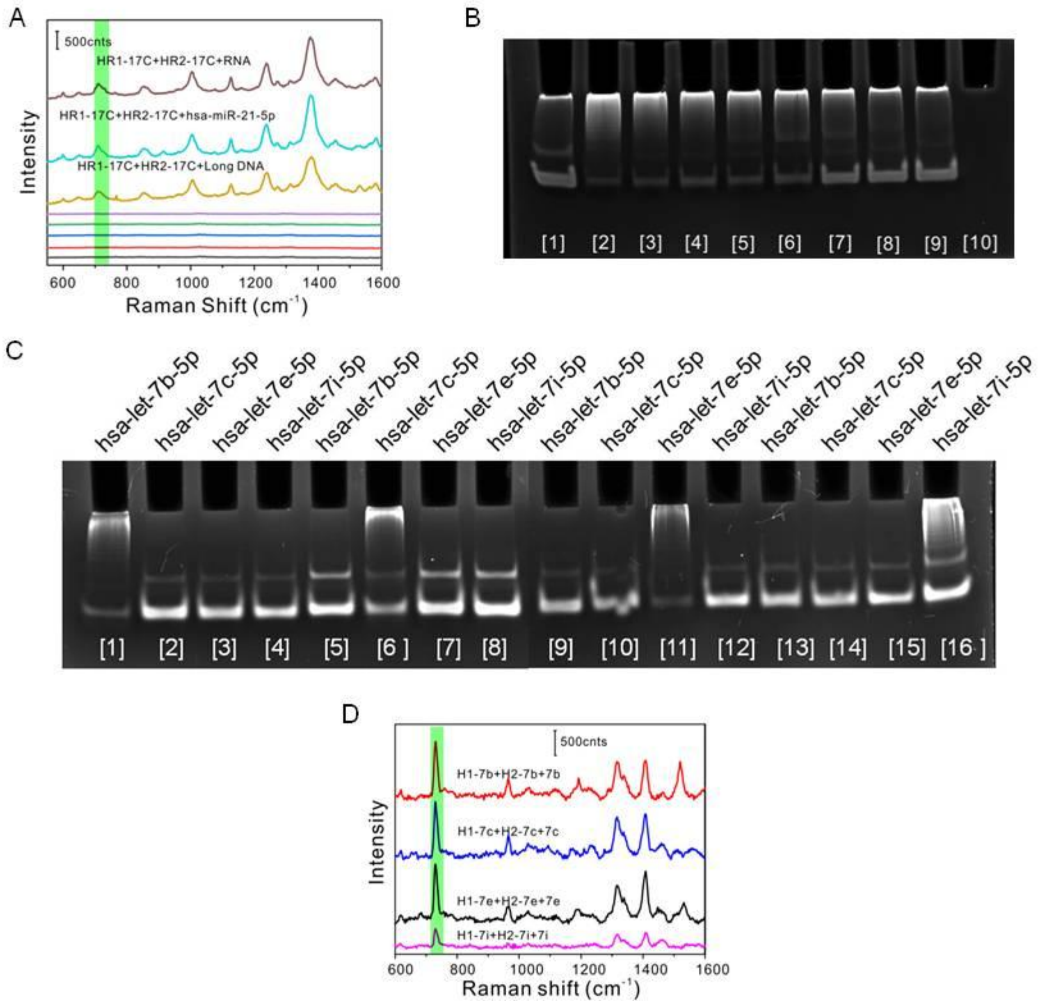

Supplementary Figure S14. Specific signal amplification of miRNA. (A) Hybridization products detected by using SERS. Unmarked is negative controls, including HR1-17C, HR2-17C, RNA and bank. The marker band of adenine around 732 cm-1 appears. (B) The detection of hsa-miR-21-5p from nine patients. Lane 10: blank. (C) Native polyacrylamide gel electrophoresis assay for detection of highly similar miRNA. Lane1-4: H1-7b and H2-7b were used as signal amplifiers; lane5-8: H1-7c and H2-7c were used as signal amplifiers; lane9-12: H1-7e and H2-7e were used as signal amplifiers; and lane13-16: H1-7i and H2-7i were used as signal amplifiers. The target could trigger the hybridization reaction only in the corresponding design system. (D) SERS showed the hybridization products. The green band indicated the marker of adenine at approximately 732 cm−1. Spectra of H1-7b+H2-7b+7b, H1-7c+H2-7c+7c, H1-7e+H2-7e+7e, H1-7i+H2-7i+7i correspond to lane 1, 6, 11 and 16 in panel C respectively.

Supplementary Table S1. Evolution of the hairpin based on Dirks and Pierce's design

Name	Strand sequences (5' to 3')	Free energy of secondary structure (-kcal mol^{-1})		Stem GC%
		37 ℃	25 ℃	
I	AGTCTAGGATTCGGCGTGGGTTAA			
H1-20	TTAACCCACGCCGAATCCTAGACTATCAAAGTATAGTCTAGGATTCGGCGTG	23.27	28.72	50.00
H1-19	TTAACCCACGCCGAATCCTAGACTACAAAGTTAGTCTAGGATTCGGCGTG	22.19	27.34	47.37
H1-18	TTAACCCACGCCGAATCCTAGACTCAAAGTAGTCTAGGATTCGGCGTG	21.81	26.76	55.56
H1-17	TTAACCCACGCCGAATCCTAGACCAAAGTGTCTAGGATTCGGCGTG	20.73	25.41	58.52
H1-15	TTAACCCACGCCGAATCCTAGCAAAGTCTAGGATTCGGCGTG	17.99	22.16	60.00
H1-13	TTAACCCACGCCGAATCCTCAAAGTAGGATTCGGCGTG	15.93	19.58	61.54
H1-11	TTAACCCACGCCGAATCCAAAGTGATTCGGCGTG	13.01	16.15	63.64
H1-10	TTAACCCACGCCGAATCAAAGTATTCGGCGTG	11.51	14.40	60.00
H1-9	TTAACCCACGCCGAACAAAGTTTCGGCGTG	10.43	13.03	66.67
H1-7	TTAACCCACGCCGCAAAGTCGGCGTG	8.53	10.66	85.71
H2-20	ATAGTCTAGGATTCGGCGTGGGTTAACACGCCGAATCCTAGACTATACTTTG	23.68	28.93	50.00
H2-19	TAGTCTAGGATTCGGCGTGGGTTAACACGCCGAATCCTAGACTAACTTTG	22.93	27.92	47.37
H2-18	AGTCTAGGATTCGGCGTGGGTTAACACGCCGAATCCTAGACTACTTTG	22.22	26.97	55.56
H2-17	GTCTAGGATTCGGCGTGGGTTAACACGCCGAATCCTAGACACTTTG	21.33	26.10	58.82
H2-15	CTAGGATTCGGCGTGGGTTAACACGCCGAATCCTAGACTTTG	18.69	22.78	60.00
H2-13	AGGATTCGGCGTGGGTTAACACGCCGAATCCTACTTTG	16.34	19.80	61.54
H2-11	GATTCGGCGTGGGTTAACACGCCGAATCACTTTG	13.61	16.85	58.33
H2-10	ATTCGGCGTGGGTTAACACGCCGAATACTTTG	11.92	14.62	60.00
H2-9	TTCGGCGTGGGTTAACACGCCGAAACTTTG	10.68	13.14	66.67
H2-7	CGGCGTGGGTTAACACGCCGACTTTG	9.23	11.27	85.71

The underlined bases at the end constituted the exposed toehold, and the blue ones at the middle constituted the sequestered toehold. The red value is the free energy of the leakage sequence. I is the initiator sequence.

Supplementary Table S2. Evolution of the hairpin based on reversed stem

Name	Strand sequences (5' to 3')	Free energy of secondary structure (-kcal mol^{-1})		Stem GC%
		37°C	25 °C	
I-b	GTGCGGCTTAGGATCTGAGGTTAA			
H1b-20	TTAACCTCAGATCCTAAGCCGCACATCAAAGTATGTGCGGCTTAGGATCTGA	23.90	29.10	50.00
H1b-19	TTAACCTCAGATCCTAAGCCGCACACAAAGTTGTGCGGCTTAGGATCTGA	22.82	27.72	52.63
H1b-18	TTAACCTCAGATCCTAAGCCGCACCAAAGTGTGCGGCTTAGGATCTGA	21.77	26.43	55.55
H1b-17	TTAACCTCAGATCCTAAGCCGCACAAAGTTGCGGCTTAGGATCTGA	19.93	24.29	52.94
H1b-16	TTAACCTCAGATCCTAAGCCGCCAAAGTGCGGCTTAGGATCTGA	18.88	23.00	56.25
H1b-15	TTAACCTCAGATCCTAAGCCGCAAAGTCGGCTTAGGATCTGA	16.64	20.50	53.33
H1b-14	TTAACCTCAGATCCTAAGCCCAAAGTGGCTTAGGATCTGA	14.47	17.97	50.00
H1b-12	TTAACCTCAGATCCTAAGCAAAGTCTTAGGATCTGA	10.39	13.39	41.67
H1b-10	TTAACCTCAGATCCTACAAAGTTAGGATCTGA	7.71	10.13	40.00
H2b-20	ATGTGCGGCTTAGGATCTGAGGTTAATCAGATCCTAAGCCGCACATACTTTG	24.23	29.44	50.00
H2b-19	TGTGCGGCTTAGGATCTGAGGTTAATCAGATCCTAAGCCGCACAACTTTG	22.99	28.03	52.63
H2b-18	GTGCGGCTTAGGATCTGAGGTTAATCAGATCCTAAGCCGCACACTTTG	22.29	27.25	55.56
H2b-17	TGCGGCTTAGGATCTGAGGTTAATCAGATCCTAAGCCGCAACTTTG	20.10	24.59	52.94
H2b-16	GCGGCTTAGGATCTGAGGTTAATCAGATCCTAAGCCGCACTTTG	19.40	23.82	56.25
H2b-15	CGGCTTAGGATCTGAGGTTAATCAGATCCTAAGCCGACTTTG	17.26	21.23	53.33
H2b-14	GGCTTAGGATCTGAGGTTAATCAGATCCTAAGCCACTTTG	14.99	18.79	50.00
H2b-12	CTTAGGATCTGAGGTTAATCAGATCCTAAGACTTTG	11.01	14.13	41.67
H2b-10	TAGGATCTGAGGTTAATCAGATCCTAACTTTG	8.37	10.83	40.00

The stem regions of DNA hairpins based on Dirks and Pierce's original design were reversed. The underlined bases at the end constituted the exposed toehold, and the blue ones at the middle constituted the sequestered toehold. The red value is the free energy of the leakage sequence. I-b is the initiator sequence.

Supplementary Table S3. Evolution of the other hairpin based on Dirks and Pierce's

Name	Strand sequences (5' to 3')	Free energy of secondary Structure at 37 °C (-kcal mol^{-1})	Stem GC%
H1C-18	TAACAAGAAAGCCAAACCAGAAGAGGTTTGGCTTTCTTGTTACCTGAT	19.70	38.89
H1C-18A	TAACAAGAAAGCCAAACCCACACAGGTTTGGCTTTCTTGTTACCTGAT	19.50	38.89
H1C-18B	TAACAAGAAAGCCAAACCTCTCTCGGTTTGGCTTTCTTGTTACCTGAT	19.30	38.89
H1C-16	ACAAGAAAGCCAAACCAGAAGAGGTTTGGCTTTCTTGTCCTGAT	17.60	43.75
H1C-16A	ACAAGAAAGCCAAACCCACACAGGTTTGGCTTTCTTGTCCTGAT	17.50	43.75
H1C-16B	ACAAGAAAGCCAAACCTCTCTCGGTTTGGCTTTCTTGTCCTGAT	17.30	43.75
H1C-15	CAAGAAAGCCAAACCAGAAGAGGTTTGGCTTTCTTGCCTGAT	16.25	46.67
H1C-14	AAGAAAGCCAAACCAGAAGAGGTTTGGCTTTCTTCCTGAT	14.71	42.86
H1C-14A	AAGAAAGCCAAACCCACACAGGTTTGGCTTTCTTCCTGAT	14.61	42.86
H1C-14B	AAGAAAGCCAAACCTCTCTCGGTTTGGCTTTCTTCCTGAT	14.41	42.86
H1C-13	AGAAAGCCAAACCAGAAGAGGTTTGGCTTTCTCCTGAT	13.71	46.15
H1C-13A	AGAAAGCCAAACCCACACAGGTTTGGCTTTCTCCTGAT	13.61	46.15
H1C-13B	AGAAAGCCAAACCTCTCTCGGTTTGGCTTTCTCCTGAT	13.41	46.15
H2C-18	TCTTCTGGTTTGGCTTTCTTGTTAATCAGGTAACAAGAAAGCCAAACC	18.98	38.89
H2C-18A	TGTGTGGGTTTGGCTTTCTTGTTAATCAGGTAACAAGAAAGCCAAACC	19.20	38.89
H12-18B	GAGAGAGGTTTGGCTTTCTTGTTAATCAGGTAACAAGAAAGCCAAACC	19.22	38.89
H2C-16	TCTTCTGGTTTGGCTTTCTTGTATCAGGACAAGAAAGCCAAACC	17.67	43.75
H2C-16A	TGTGTGGGTTTGGCTTTCTTGTATCAGGACAAGAAAGCCAAACC	17.62	43.75
H2C-16B	GAGAGAGGTTTGGCTTTCTTGTATCAGGACAAGAAAGCCAAACC	17.64	43.75
H2C-15	TCTTCTGGTTTGGCTTTCTTGATCAGGCAAGAAAGCCAAACC	16.53	46.67
H2C-14	TCTTCTGGTTTGGCTTTCTTATCAGGAAGAAAGCCAAACC	14.78	42.86
H2C-14A	TGTGTGGGTTTGGCTTTCTTATCAGGAAGAAAGCCAAACC	14.73	42.86
H2C-14B	GAGAGAGGTTTGGCTTTCTTATCAGGAAGAAAGCCAAACC	14.75	42.86
H2C-13	TCTTCTGGTTTGGCTTTCTATCAGGAGAAAGCCAAACC	13.78	46.15
H2C-13A	TGTGTGGGTTTGGCTTTCTATCAGGAGAAAGCCAAACC	13.73	46.15
H2C-13B	GAGAGAGGTTTGGCTTTCTATCAGGAGAAAGCCAAACC	13.75	46.15

The other hairpin based on Dirks and Pierce's were designed. The underlined bases at the end constituted the exposed toehold, and the blue ones at the middle constituted the sequestered toehold. The red value is the free energy of the leakage sequence.

Supplementary Table S4. Evolution of the other DNA Hairpin sequences

Name	Strand sequences (5' to 3')	Free energy of secondary structure (-kcal mol^{-1})		Stem GC%
		37 ℃	25 ℃	
hsa-miR-21-5p	UAGCUUAUCAGACUGAUGUUGA			
HR1-17C	TCAACATCAGTCTGATAAGCTACCAAAGTGTAGCTTATCAGACTGA	17.23	21.67	41.17
HR1-17T	TCAACATCAGTCTGATAAGCTATCAAAGTATAGCTTATCAGACTGA	16.47	20.91	35.29
HR1-16	TCAACATCAGTCTGATAAGCTACAAAGTTAGCTTATCAGACTGA	15.39	19.53	37.50
HR1-15	TCAACATCAGTCTGATAAGCTCAAAGTAGCTTATCAGACTGA	15.01	18.94	40.00
HR1-14	TCAACATCAGTCTGATAAGCCAAAGTGCTTATCAGACTGA	13.93	17.59	42.86
HR1-13	TCAACATCAGTCTGATAAGCAAAGTCTTATCAGACTGA	11.69	15.10	38.46
HR1-12	TCAACATCAGTCTGATAACAAAGTTTATCAGACTGA	10.01	13.10	33.33
HR1-11	TCAACATCAGTCTGATACAAAGTTATCAGACTGA	9.01	11.83	36.36
HR2-17T	ATAGCTTATCAGACTGATGTTGATCAGTCTGATAAGCTATACTTTG	17.73	22.26	35.29
HR2-17C	GTAGCTTATCAGACTGATGTTGATCAGTCTGATAAGCTACACTTTG	18.68	23.51	41.18
HR2-16	TAGCTTATCAGACTGATGTTGATCAGTCTGATAAGCTAACTTTG	16.98	21.25	37.50
HR2-15	AGCTTATCAGACTGATGTTGATCAGTCTGATAAGCTACTTTG	16.27	20.30	40.00
HR2-14	GCTTATCAGACTGATGTTGATCAGTCTGATAAGCACTTTG	15.38	19.43	42.86
HR2-13	CTTATCAGACTGATGTTGATCAGTCTGATAAGACTTTG	13.24	16.85	38.46
HR2-12	TTATCAGACTGATGTTGATCAGTCTGATAAACTTTG	11.11	14.35	33.33
HR2-11	TATCAGACTGATGTTGATCAGTCTGATACTTTG	10.60	13.55	36.36
hsa-let-7a-5p	UGAGGUAGUAGGUUGUAUAGUU			
H1-7a-16	AACTAT ACAACCTACTACCTCACAAAGTTGAGGTAGTAGGTTGT	16.60		43.75
H1-7a-15	AACTAT ACAACCTACTACCTCCAAAGTGAGGTAGTAGGTTGT	15.55		46.67
H1-7a-14	AACTAT ACAACCTACTACCTCAAAGTAGGTAGTAGGTTGT	14.06		42.86
H1-7a-13	AACTAT ACAACCTACTACCCAAAGTGGTAGTAGGTTGT	12.97		46.15
H2-7a-16	TGAGGTAGTAGGTTGTATAGTTACAACCTACTACCTCAACTTTG	17.19		43.75
H2-7a-15	GAGGTAGTAGGTTGTATAGTTACAACCTACTACCTCACTTTG	16.49		46.67
H2-7a-14	AGGTAGTAGGTTGTATAGTTACAACCTACTACCTACTTTG	14.80		42.86
H2-7a-13	GGTAGTAGGTTGTATAGTTACAACCTACTACCACTTTG	13.91		46.15
hsa-miR-18a-5p	UAAGGUGCAUCUAGUGCAGAUAG			
H1-18a-15	TATCTG CACTAGATGCACCTTCAAAGTAAGGTGCATCTAGTG	16.13		46.67
H1-18a-14	TATCTG CACTAGATGCACCTCAAAGTAGGTGCATCTAGTG	15.13		50.0
H1-18a-13	TATCTG CACTAGATGCACCCAAAGTGGTGCATCTAGTG	14.05		53.85

H1-18a-12	TATCTG CACTAGATGCACCAAAGTGTGCATCTAGTG	12.21		50.0
H2-18a-15	AAGGTGCATCTAGTGCAGATACACTAGATGCACCTTACTTTG	16.34		46.67
H2-18a-14	AGGTGCATCTAGTGCAGATACACTAGATGCACCTACTTTG	15.34		50.0
H2-18a-13	GGTGCATCTAGTGCAGATACACTAGATGCACCACTTTG	14.45		53.85
H2-18a-12	GTGCATCTAGTGCAGATACACTAGATGCACACTTTG	12.61		50.0
hsa-miR-20a-5p	UAAAGUGCUUAUAGUGCAGGUAG			
H1-20a-17	CTACCTGCACTATAAGCACTTTACAAAGTTAAAGTGCTTATAGTGC	16.63		35.29
H1-20a-15	CTACCT GCACTATAAGCACTTCAAAGTAAGTGCTTATAGTGC	15.25		40.0
H2-20a-17	AAAGTGCTTATAGTGCCAGGTAGCACTATAAGCACTTTACTTTG	17.08		35.29
H2-20a-15	AAGTGCTTATAGTGCCAGGTAGCACTATAAGCACTTACTTTG	15.37		40.0
hsa-miR-29a-3p	UAGCACCAUCUGAAAUCGGUUA			
H1-29a-16	AACCGATTTCAGATGGTGCTAACAAAGTTTAGCACCATCTGAAA	16.24		37.5
H1-29a-15	AACCGATTTCAGATGGTGCTACAAAGTTAGCACCATCTGAAA	15.24		40.0
H2-29a-16	TTAGCACCATCTGAAATCGGTTTTTCAGATGGTGCTAAACTTTG	15.96		37.5
H2-29a-15	TAGCACCATCTGAAATCGGTTTTTCAGATGGTGCTAACTTTG	15.45		40.0
hsa-miR-30a-5p	UGUAAACAUCCUCGACUGGAAG			
H1-30a-16	CTTCCAGTCGAGGATGTTTACACAAAGTTGTAAACATCCTCGAC	16.75		43.75
H1-30a-15	CTTCCAGTCGAGGATGTTTACCAAAGTGTAAACATCCTCGAC	15.70		46.67
H2-30a-16	TGTAAACATCCTCGACTGGAAGGTCGAGGATGTTTACAACTTTG	18.26		43.75
H2-30a-15	GTAAACATCCTCGACTGGAAGGTCGAGGATGTTTACAACTTTG	17.56		46.67
hsa-miR-95-3p	UUCAACGGGUAUUUAUUGAGCA			
H1-95-16	TGCTCA ATAAATACCCGTTGAACAAAGTTTCAACGGGTATTTAT	15.16		31.25
H2-95-16	TTCAACGGGTATTTATTGAGCAATAAATACCCGTTGAAACTTTG	16.97		31.25
hsa-miR-181a-5p	AACAUUCAACGCUGUCGGUGAGU			
H1-181a-15	ACTCACCGACAGCGTTGAATGCAAAGTCATTCAACGCTGTCG	17.79		53.33
H1-181a-13	ACTCACCGACAGCGTTGAACAAAGTTTCAACGCTGTCG	15.06		53.85
H2-181a-15	CATTCAACGCTGTCGGTGAGTCGACAGCGTTGAATGACTTTG	18.41		53.33
H2-181a-13	TTCAACGCTGTCGGTGAGTCGACAGCGTTGAAACTTTG	15.23		53.85
hsa-miR-302a-3p	UAAGUGCUUCCAUGUUUUGGUGA			
H1-302a-15	TCACCAAAACATGGAAGCACTCAAAGTAGTGCTTCCATGTTT	16.01		40.0
H1-302a-14	TCACCAAAACATGGAAGCACCAAAGTGTGCTTCCATGTTT	14.93		42.86

H2-302a-15	AGTGCTTCCATGTTTTGGTGAAAACATGGAAGCACTACTTTG	17.38		40.0
H2-302a-14	GTGCTTCCATGTTTTGGTGAAAACATGGAAGCACACTTTG	16.49		42.86

The other DNA Hairpin sequences based on miRNA were designed.The underlined bases at the end constituted the exposed toehold, and the blue ones at the middle constituted the sequestered toehold. The red value is the free energy of the leakage sequence. miRNA sequences were from the datebase of miRTarBase: (http://mirtarbase.mbc.nctu.edu.tw/php/index.php）

Supplementary Table S5. Evolution of the hairpin based on changed toehold

Name	Strand sequences (5' to 3')	Free energy of secondary Structure at 37 °C (-kcal mol^{-1})	Stem GC%
I-18a	TAAGGTGCATCTAGTGCAGATAG		
H1-18a-18b	CTATCTGCACTAGATGCACCTTACGATACGGTAAGGTGCATCTAGTGC	20.68	50.0
H1-18a-17b	CTATCTGCACTAGATGCACCTTAGATACGTAAGGTGCATCTAGTGC	18.74	47.06
H1-18a-15b	CTATCTGCACTAGATGCACCTGATACGAGGTGCATCTAGTGC	17.16	53.33
H1-18a-14b	CTATCTGCACTAGATGCACCGATACGGGTGCATCTAGTGC	16.38	57.14
H1-18a-13b	CTATCTGCACTAGATGCACGATACGGTGCATCTAGTGC	14.54	53.85
H1-18a-12b	CTATCTGCACTAGATGCAGATACGTGCATCTAGTGC	12.6	50.0
H1-18a-11b	CTATCTGCACTAGATGCGATACGGCATCTAGTGC	11.65	54.55
H1-18a-10b	CTATCTGCACTAGATGGATACGCATCTAGTGC	9.51	50.0
H2-18a-18b	GTAAGGTGCATCTAGTGCAGATAGGCACTAGATGCACCTTACCGTATC	20.38	50.0
H2-18a-17b	TAAGGTGCATCTAGTGCAGATAGGCACTAGATGCACCTTACGTATC	19.19	47.06
H2-18a-15b	AGGTGCATCTAGTGCAGATAGGCACTAGATGCACCTCGTATC	17.19	53.33
H2-18a-14b	GGTGCATCTAGTGCAGATAGGCACTAGATGCACCCGTATC	16.08	57.14
H2-18a-13b	GTGCATCTAGTGCAGATAGGCACTAGATGCACCGTATC	14.24	53.85
H2-18a-12b	TGCATCTAGTGCAGATAGGCACTAGATGCACGTATC	12.47	50.0
H2-18a-11b	GCATCTAGTGCAGATAGGCACTAGATGCCGTATC	11.35	54.55
H2-18a-10b	CATCTAGTGCAGATAGGCACTAGATGCGTATC	9.03	50.0
I-181a	AACATTCAACGCTGTCGGTGAGT		
H1-181a-17b	ACTCACCGACAGCGTTGAATGTTCCATACAACATTCAACGCTGTCG	19.73	47.06
H1-181a-15b	ACTCACCGACAGCGTTGAATGCCATACCATTCAACGCTGTCG	17.69	53.33
H1-181a-14b	ACTCACCGACAGCGTTGAATCCATACATTCAACGCTGTCG	15.84	50.0
H1-181a-13b	ACTCACCGACAGCGTTGAACCATACTTCAACGCTGTCG	14.96	53.85
H1-181a-11b	ACTCACCGACAGCGTTGCCATACCAACGCTGTCG	13.06	63.64
H1-181a-10b	ACTCACCGACAGCGTTCCATACAACGCTGTCG	11.21	60.0
H1-181a-9b	ACTCACCGACAGCGTCCATACACGCTGTCG	10.21	66.67
H1-181a-8b	ACTCACCGACAGCGCCATACCGCTGTCG	9.17	75.0
H2-181a-17b	AACATTCAACGCTGTCGGTGAGTCGACAGCGTTGAATGTTGTATGG	20.38	47.06
H2-181a-15b	CATTCAACGCTGTCGGTGAGTCGACAGCGTTGAATGGTATGG	17.93	53.33
H2-181a-14b	ATTCAACGCTGTCGGTGAGTCGACAGCGTTGAATGTATGG	16.49	50.0
H2-181a-13b	TTCAACGCTGTCGGTGAGTCGACAGCGTTGAAGTATGG	15.12	53.85

H2-181a-11b	CAACGCTGTCGGTGAGTCGACAGCGTTGGTATGG	13.30	63.64
H2-181a-10b	AACGCTGTCGGTGAGTCGACAGCGTTGTATGG	11.86	60.0
H2-181a-9b	ACGCTGTCGGTGAGTCGACAGCGTGTATGG	10.86	66.67
H2-181a-8b	CGCTGTCGGTGAGTCGACAGCGGTATGG	9.41	75.0

The DNA Hairpin sequences based on changed toehold were designed. The underlined bases at the end constituted the exposed toehold, and the blue ones at the middle constituted the sequestered toehold. The red value is the free energy of the leakage sequence. I-18a is the corresponding DNA sequence of hsa-miR-18a-5p. I-181a is the corresponding DNA sequence of hsa-miR-181a-5p.

Supplementary Table S6. Modified sequences in stem region by mutation and substitution

Name	Strand sequences (5' to 3')	Free energy of secondary Structure at 37 °C (-kcal mol^{-1})
H1b-17	TTAACCTCAGATCCTAAGCCGCACAAAGTTGCGGCTTAGGATCTGA	19.93
M-1	TTAACC TAAGATCCTAAGCCGCACAAAGTTGCGGCTTAGGATCTGA	17.94
M-2	TTAACC TCAGATCCAAAGCCGCACAAAGTTGCGGCTTAGGATCTGA	16.95
M-3	TTAACC TCAGATCCTAAGCCGAACAAAGTTGCGGCTTAGGATCTGA	16.44
M-4	TTAACC TCAGATCCTAAGCCGATCAAAGTTGCGGCTTAGGATCTGA	16.44
M-5	TTAACC TAAGATCCAAAGCCGCACAAAGTTGCGGCTTAGGATCTGA	14.96
M-6	TTAACC TCAGATACTATGCCGCACAAAGTTGCGGCTTAGGATCTGA	14.04
M-7	TTAACC TAAGATCCTAAGCCGAACAAAGTTGCGGCTTAGGATCTGA	14.45
M-8	TTAACC TCAGATCCAAAGCCGAACAAAGTTGCGGCTTAGGATCTGA	13.46
H1-15	TTAACCCACGCCGAATCCTAGCAAAGTCTAGGATTCGGCGTG	17.99
H1-15- 1	TTAACCAACGCCGAATCCTAACAAAGTTTAGGATTCGGCGTT	16.71
H1-15- 2	TTAACCCACGCCGAATAATCAAAGCTATTATTCGGCGTG	16.03
H1-15- 3	TTAACCCACGAAGAATCCTAGCAAAGTCTAGGATTCTTCGTG	15.32
H1-15- 4	TTAACCCAAGCAGAATCCTAGCAAAGTCTAGGATTCTGCTTG	15.38
H1-15- 5	TTAACCCACGAAAGAATCCTAGCAAAGTCTAGGATTCTTTCGTG	16.32
H1-15- 6	TTAACCCACGAAGAATCACTAGCAAAGTCTAGTGATTCTTCGTG	16.37
H1-15- 7	TTAACCCAAGACAGAATCCTAGCAAAGTCTAGGATTCTGTCTTG	15.88
H1-15- 8	TTAACCCAAGCAGAATCCTAAGCAAAGTCTTAGGATTCTGCTTG	16.38
H1-15-9	TTAACC AACGAAGAATCATAGCAAAGTCTATGATTCTTCGTT	13.93
H1-15- 10	TTAACC CAAACAGAATCCTAACAAAGTTTAGGATTCTGTTTG	13.62
H2-15	CTAGGATTCGGCGTGGGTTAACACGCCGAATCCTAGACTTTG	18.69
H2-15-1	TTAGGATTCGGCGTTGGTTAAAACGCCGAATCCTAAACTTTG	16.71
H2-15-2	CTATTATTCGGCGTGGGTTAACACGCCGAATAATAGACTTTG	16.73
H2-15-3	CTAGGATTCTTCGTGGGTTAACACGAAGAATCCTAGACTTTG	16.02
H2-15-4	CTAGGATTCTGCTTGGGTTAACAAGCAGAATCCTAGACTTTG	16.08
H2-15-5	CTAGGATTCTTTCGTGGGTTAACACGAAAGAATCCTAGACTTTG	17.02
H2-15-6	CTAGTGATTCTTCGTGGGTTAACACGAAGAATCACTAGACTTTG	17.07
H2-15-7	CTAGGATTCTGTCTTGGGTTAACAAGACAGAATCCTAGACTTTG	16.58
H2-15-8	CTTAGGATTCTGCTTGGGTTAACAAGCAGAATCCTAAGACTTTG	17.08
H2-15-9	CTATGATTCTTCGTTGGTTAAAACGAAGAATCATAGACTTTG	14.38

H2-15-10	TTAGGATTCTGTTTGGGTTAACAAACAGAATCCTAAACTTTG	13.87
H1-18a-17b	CTATCTGCACTAGATGCACCTTAGATACGTAAGGTGCATCTAGTGC	18.74
H1-18a-01	CTATCTGCACTAGATGCACCTATGATACGTAAGGTGCATCTAGTGC	17.06
H1-18a-02	CTATCTGCACTAGATGCACCAATGATACGTAAGGTGCATCTAGTGC	15.98
H1-18a-03	CTATCTGCAATAGATGCAACTGATACGAGTTGCATCTATTGC	15.48
H2-18a-03	AGTTGCATCTATTGCAGATAGGCAATAGATGCAACTCGTATC	15.51
HR1-16	TCAACATCAGTCTGATAAGCTACAAAGTTAGCTTATCAGACTGA	15.39
HR1-16-01	TCAACATCCGTCTGATAAGCTACAAAGTTAGCTTATCAGACGGA	16.67
HR1-16-02	TCAACATCAGTCCGATAAGCTACAAAGTTAGCTTATCGGACTGA	16.67
HR1-16-03	TCAACATCAGTCTGATGAGCTACAAAGTTAGCTCATCAGACTGA	16.56
HR1-16-04	TCAACA TCAGTCTGATAAGCTCCAAAGTGAGCTTATCAGACTGA	16.51
HR2-16	TAGCTTATCAGACTGATGTTGATCAGTCTGATAAGCTAACTTTG	16.98
HR2-16-01	TAGCTTATCAGACGGATGTTGATCCGTCTGATAAGCTAACTTTG	18.26
HR2-16-02	TAGCTTATCGGACTGATGTTGATCAGTCCGATAAGCTAACTTTG	18.26
HR2-16-03	TAGCTCATCAGACTGATGTTGATCAGTCTGATGAGCTAACTTTG	18.15
HR2-16-04	GAGCTTATCAGACTGATGTTGATCAGTCTGATAAGCTCACTTTG	17.96

The changed nucleotides were marked in red. The red value is the free energy of the leakage sequence.

Supplementary Table S7. DNA sequences used in effect of toehold and loop length on DNA assembly behaviour

Name	Strand sequences (5' to 3')	Free energy of secondary Structure at 37 °C (-kcal mol^{-1})
H1b-17-8T	TTAACCTCAGATCCTAAGCCGCACATTGCAAAGTATGTGCGGCTTAGGATCT	20.82
H2b-17-8T	ATGTGCGGCTTAGGATCTGAGGTTAAAGATCCTAAGCCGCACATACTTTGCA	21.18
H1-17-8T	TTAACCCACGCCGAATCCTAGACTATGTCAAAGTATAGTCTAGGATTCGGCG	20.52
H2-17-8T	ATAGTCTAGGATTCGGCGTGGGTTAACGCCGAATCCTAGACTATACTTTGAC	21.83
H1-18a-17b-8T	TTCTATCTGCACTAGATGCACCTTAAGATACGATAAGGTGCATCTAGTGC	18.74
H2-18a-17b-8T	TAAGGTGCATCTAGTGCAGATAGAAGCACTAGATGCACCTTATCGTATCT	18.99
HR1-17C-8T	CATCAACATCAGTCTGATAAGCTACCAAAGTCCGTAGCTTATCAGACTGA	16.73
HR2-17C-8T	GTAGCTTATCAGACTGATGTTGATGTCAGTCTGATAAGCTACGGACTTTG	16.52
H1b-17	TTAACCTCAGATCCTAAGCCGCACAAAGTTGCGGCTTAGGATCTGA	19.93
E1	TCAGATCCTAAGCCGCACcCAAAGTTGCGGCTTAGGATCTGA	19.63
E3	TCAGATCCTAAGCCGCActaCAAAGTTGCGGCTTAGGATCTGA	19.63
E5	TCAGATCCTAAGCCGCActccaCAAAGTTGCGGCTTAGGATCTGA	19.53
E7	TCAGATCCTAAGCCGCActcaatcCAAAGTTGCGGCTTAGGATCTGA	19.33
E9	TCAGATCCTAAGCCGCActacatccaCAAAGTTGCGGCTTAGGATCTGA	19.23
E12	TCAGATCCTAAGCCGCActacatacaccaCAAAGTTGCGGCTTAGGATCTGA	18.93
E15	TCAGATCCTAAGCCGCActacatcacataccaCAAAGT TGCGGCTTAGGATCTGA	18.73
E18	TCAGATCCTAAGCCGCActacatacatacataccaCAAAGT TGCGGCTTAGGATCTGA	18.33
T-01	TCAGATCCTAAGCCGCAACTTTG	
T-02	TCCTAAGCCGCAACTTTG	
H1-18a-17b	CTATCTGCACTAGATGCACCTTAGATACGTAAGGTGCATCTAGTGC	18.74
18a-E1	GCACTAGATGCACCTTAaGATACGTAAGGTGCATCTAGTGC	17.98
18a-E3	GCACTAGATGCACCTTAataGATACGTAAGGTGCATCTAGTGC	17.93
18a-E5	GCACTAGATGCACCTTAactcaGATACGTAAGGTGCATCTAGTGC	17.83
18a-E7	GCACTAGATGCACCTTAactcattGATACGTAAGGTGCATCTAGTGC	17.63
18a-E9	GCACTAGATGCACCTTAactacactaGATACGTAAGGTGCATCTAGTGC	17.53
18a-E12	GCACTAGATGCACCTTAactacactacacGATACGTAAGGTGCATCTAGTGC	17.23
18a-E15	GCACTAGATGCACCTTAactacactacactacGATACGTAAGGTGCATCTAGTGC	17.03
18a-E18	GCACTAGATGCACCTTAactacactacactacactGATACGTAAGGTGCATCTAGTGC	16.63

T-18a-01	GCACTAGATGCACCTTACGTATC	
T-18a-02	AGATGCACCTTACGTATC	
H1-181a-17b	ACTCACCGACAGCGTTGAATGTTCCATACAACATTCAACGCTGTCG	
181a-E1	CGACAGCGTTGAATGTTcCCATACAACATTCAACGCTGTCG	18.91
181a -E3	CGACAGCGTTGAATGTTctaCCATACAACATTCAACGCTGTCG	18.91
181a-E5	CGACAGCGTTGAATGTTctacaCCATACAACATTCAACGCTGTCG	18.81
181a -E7	CGACAGCGTTGAATGTTctacataCCATACAACATTCAACGCTGTCG	18.61
181a -E9	CGACAGCGTTGAATGTTctacatacaCCATACAACATTCAACGCTGTCG	18.51
181a-E12	CGACAGCGTTGAATGTTctacatacatacCCATACAACATTCAACGCTGTCG	18.21
181a -E15	CGACAGCGTTGAATGTTctacatacatacataCCATACAACATTCAACGCTGTCG	18.01
181a -E18	CGACAGCGTTGAATGTTctacatacatacataccaCCATACAACATTCAACGCTGTCG	17.61
T-181a-01	CGACAGCGTTGAATGTTGTATGG	
T-181a-02	GCGTTGAATGTTGTATGG	

The underlined bases at the end constituted the exposed toehold, and the blue ones at the middle constituted the sequestered toehold. Inserting bases were highlighted in red.

Supplementary Table S8. DNA sequences used in effect of initiator on HCR

Name	Strand sequences (5' to 3')
H1b-17	TTAACCTCAGATCCTAAGCCGCACAAAGTTGCGGCTTAGGATCTGA
H2b-17	TGCGGCTTAGGATCTGAGGTTAATCAGATCCTAAGCCGCAACTTTG
T-1	TGCGGCTTAGGATCTGAGGTTA
T-2	TGCGGCTTAGGATCTGAGGTT
T-3	TGCGGCTTAGGATCTGAGGT
T-4	TGCGGCTTAGGATCTGAGG
T-5	GTGCGGCTTAGGATCTGAGGTTAA
T-6	GCGGCTTAGGATCTGAGGTTAA
T-7	CGGCTTAGGATCTGAGGTTAA
T-8	GGCTTAGGATCTGAGGTTAA
T-9	GGCTTAGGATCTGAGGTTAA
T-10	GCCTTAGGATCTGAGGTTAA
T-11	CCTTAGGATCTGAGGTTAA
T-12	CTTAGGATCTGAGGTTAA
H1C-18	TAACAAGAAAGCCAAACCGAGATGGGTTTGGCTTTCTTGTTACCTGAT
H2C-18	CATCTCGGTTTGGCTTTCTTGTTAATCAGGTAACAAGAAAGCCAAACC
T-18-2	ATCAGGTAACAAGAAAGCCAAA
T-18-3	ATCAGGTAACAAGAAAGCCAA
T-18-4	ATCAGGTAACAAGAAAGCCA
T-18-5	ATCAGGTAACAAGAAAGCC
T-18-6	ATCAGGTAACAAGAAAGC
T-18-7	ATCAGGTAACAAGAAAG
T-18-8	ATCAGGTAACAAGAAA
H1-18a-17b	CTATCTGCACTAGATGCACCTTAGATACGTAAGGTGCATCTAGTGC
H2-18a-17b	TAAGGTGCATCTAGTGCAGATAGGCACTAGATGCACCTTACGTATC
T-18a-3	GGTGCATCTAGTGCAGATAG
T-18a-4	GTGCATCTAGTGCAGATAG
T-18a-5	TGCATCTAGTGCAGATAG
T-18a-6	GCATCTAGTGCAGATAG
T-18a-7	CATCTAGTGCAGATAG
T-18a-8	ATCTAGTGCAGATAG

H1d-20	GTGGGAGTCGTCTGTAACATCTTCTAATGTTACAGACGACTCCCACACTGGA
H2d-20	T AGAAGATGTTACAGACGACTCCCACTCCAGTGTGGGAGTCGTCTGTAACAT
T-20-3	TCCAGTGTGGGAGTCGTCTGTAA
T-20-4	TCCAGTGTGGGAGTCGTCTGTA
T-20-5	TCCAGTGTGGGAGTCGTCTGT
T-20-6	TCCAGTGTGGGAGTCGTCTG
T-20-7	TCCAGTGTGGGAGTCGTCT
T-20-8	TCCAGTGTGGGAGTCGTC
H1E-20	AT GTTACAGACGACTCCCACTCCAGTGTGGGAGTCGTCTGTAACATGAAGTA
H2E-20	ACTGGAGTGGGAGTCGTCTGTAACATTACTTCATGTTACAGACGACTCCCAC
T-E20-4	TACTTCATGTTACAGACGACTC
T-E20-5	TACTTCATGTTACAGACGACT
T-E20-6	TACTTCATGTTACAGACGAC
T-E20-7	TACTTCATGTTACAGACGA
T-E20-8	TACTTCATGTTACAGACG
T-E20-9	TACTTCATGTTACAGAC

The underlined bases at the end constituted the exposed toehold, and the blue ones at the middle constituted the sequestered toehold.

Supplementary Table S9. DNA Fairpin for detection of miRNA family

Name	Strand sequences (5'to3')	Free energy of secondary structure at 37 °C (-kcal mol^{-1})
hsa-let-7d-5p	AGAGGUAGUAGGUUGCAUAGUU	
H1-7d-0	AACTATGCAACCTACTACCTCTCAAAGTAGAGGTAGTAGGTTGC	17.38
H2-7d-0	AGAGGTAGTAGGTTGCTAAGTTGCAACCTACTACCTCTACTTTG	17.60
H1-7d	AACTAT GCAACCTACTACCTCTACCAAAGTGTAGAGGTAGTAGGTTGC	19.60
H2-7d	GTAGAGGTAGTAGGTTGCTAAGTTGCAACCTACTACCTCTACACTTTG	20.01
hsa-let-7b-5p	UGAGGUAGUAGGUUGUGUGGUU	
H1-7b-0	AGTAGGTTGTGTGGTTCAAAGTAACCACACAACCTACTACCTCA	17.30
H2-7b-0	ACTTTGAACCACACAACCTACTTGAGGTAGTAGGTTGTGTGGTT	17.24
H1-7b	AGTAGGTTGTGTGGTTGTCCAAAGTGACAACCACACAACCTACTACCTCA	21.69
H2-7b	ACTTTGGACAACCACACAACCTACTTGAGGTAGTAGGTTGTGTGGTTGTC	21.42
hsa-let-7a-5p	UGAGGUAGUAGGUUGUAUAGUU	
H1-7a-0	AACTATACAACCTACTACCTCACAAAGTTGAGGTAGTAGGTTGT	16.60
H2-7a-0	TGAGGTAGTAGGTTGTTGAGGTACAACCTACTACCTCAACTTTG	16.01
H1-7a	AACTATACAACCTACTACCTCACTCCAAAGTGAGTGAGGTAGTAGGTTGT	21.02
H2-7a	GAGTGAGGTAGTAGGTTGTTGAGGTACAACCTACTACCTCACTCACTTTG	20.78
hsa-let-7c-5p	UGAGGUAGUAGGUUGUAUGGUU	
H1-7c-0	ATAGTAGGTTGTATGGTTCAAAGTAACCATACAACCTACTACCTCA	17.30
H2-7c-0	ACTTTG AACCATACAACCTACTTGAGGTATAGTAGGTTGTATGGTT	17.24
H1-7c	ATAGTAGGTTGTATGGTTCACGCAAAGTCGTGAACCATACAACCTACTACCTCA	21.42
H2-7c	ACTTTG CGTGAACCATACAACCTACT TGAGGT ATAGTAGGTTGTATGGTTCACG	22.02
hsa-let-7e-5p	UGAGGUAGGAGGUUGUAUAGUU	
H1-7e-0	AACTATACAACCTCCTACCTCACAAAGTTGAGGTAGGAGGTTGT	15.56
H2-7e-0	AACTATACAACCTCCTACCTCATGAGGTACAACCTCCTACCTCA ACTTTG	15.50
H1-7e	AACTATACAACCTCCTACCTCAGGCCAAAGTGCCTGAGGTAGGAGGTTGT	21.69
H2-7e	GCCAACTATACAACCTCCTTGAGGTACAACCTCCTACCTCAGGCACTTTG	21.42
hsa-let-i-5p	UGAGGUAGUAGUUUGUGCUGUU	
H1-7i-0	AGTAGTTTGTGCTGTTCAAAGTAACAGCACAAACTACTACCTCA	16.70
H2-7i-0	ACTTTGAACAGCACAAACTACTTGAGGTAGTAGTTTGTGCTGTT	16.64
H1-7i	AGTAGTTTGTGCTGTTGCCAAAGTGCAACAGCACAAACTACTACCTCA	20.59
H2-7i	ACTTTGGCAACAGCACAAACTACTTGAGGTAGTAGTTTGTGCTGTTGC	20.32

The underlined bases at the middle were the loop as the sequestered toehold and those at the end constituted the exposed toehold. Added bases were highlighted in red.

References

1. Ivani, I., Dans, P. D., Noy, A., et al. (2016) Parmbsc1: a refined force field for DNA simulations. *Nature methods*, **13**, 55-58.

2. Pérez, A., Marchán. I., Svozil, D., et al. (2007) Refinement of the AMBER force-field for nucleic acid simulations: Improving the representation of a/c conformations. *Biophysical J*, **92**, 3817-3829.
